# Profiling Hepatic microRNAs in Zebrafish: Fluoxetine Exposure Mimics a Fasting Response That Targets AMP-Activated Protein Kinase (AMPK)

**DOI:** 10.1371/journal.pone.0095351

**Published:** 2014-04-21

**Authors:** Paul M. Craig, Vance L. Trudeau, Thomas W. Moon

**Affiliations:** Department of Biology and Centre for Advanced Research in Environmental Genomics, University of Ottawa, Ottawa, Ontario, Canada; International Centre for Genetic Engineering and Biotechnology, Italy

## Abstract

This study examined the similarities in microRNA profiles between fasted and fluoxetine (FLX) exposed zebrafish and downstream target transcripts and biological pathways. Using a custom designed microarray targeting 270 zebrafish miRNAs, we identified 9 differentially expressed miRNAs targeting transcripts in biological pathways associated with anabolic metabolism, such as adipogenesis, cholesterol biosynthesis, triacylglycerol synthesis, and insulin signaling. Exposure of female zebrafish to 540 ng/L FLX, an environmentally relevant concentration and a known metabolic repressor, increased specific miRNAs indicating greater inhibition of these pathways in spite of continued feeding. Further examination revealed two specific miRNAs, dre-let-7d and dre-miR-140-5p, were predicted *in silico* to bind to a primary regulator of metabolism, adenosine monophosphate-activated protein kinase (AMPK), and more specifically the two isoforms of the catalytic subunit, AMPK**α**1 and **α**2, respectively. Real-time analysis of the relative transcript abundance of the **α**1 and **α**2 mRNAs indicated a significant inverse relationship between specific miRNA and target transcript. This suggests that AMPK-related pathways may be compromised during FLX exposure as a result of increased miRNA abundance. The mechanism by which FLX regulates miRNA abundance is unknown but may be direct at the liver. The serotonin transporter, slc6a4, is the target of FLX and other selective serotonin reuptake inhibitors (SSRI) and it was found to be expressed in the liver, although treatment did not alter expression of this transporter. Exposure to FLX disrupts key hepatic metabolic pathways, which may be indicative of reduced overall fitness and these effects may be linked to specific miRNA abundance. This has important implications for the heath of fish because concentrations of SSRIs in aquatic ecosystems are continually increasing.

## Introduction

MicroRNAs (miRNAs) are major post-transcriptional regulators [1]–[3]. These short, noncoding RNAs regulate mRNA translation and/or stability by binding to ’seed sequences’ in the 3′UTR of a target gene. Ultimately, this binding blocks translation of the target gene at the post-transcriptional level [4]. MicroRNAs are involved in a number of diverse biological processes including metabolism, cell development and apoptosis, and are sensitive to toxicological insults, such as heavy metals (Cd or Al) and microcystins [5], [6], and to postprandial regulation [2]. Given that miRNAs are regulated by hormones and neurotransmitters [7], [8], they may also be affected by endocrine disrupting chemicals. Many human pharmaceuticals are now detected in aquatic environments and some are considered to be important endocrine disruptors [9].

The substantial prescription rates and use of human and veterinary drugs and the inability to effectively remove them from treated sewage wastewater, has led to growing concerns that the metabolically active ingredients excreted in human and animal waste will accumulate in lakes and rivers and detrimentally effect resident aquatic organisms [10]–[12]. Fluoxetine (FLX), a selective serotonin re-uptake inhibitor (SSRI) and the metabolically active ingredient of Prozac, was introduced by Eli Lilly 26 years ago, and today is amongst the most prescribed SSRIs for the treatment of depression and anxiety. FLX has been found at detectable levels in surface waters surrounding wastewater treatment plants [13], [14] and has one of the highest acute toxicity levels of any human pharmaceutical for non-target, aquatic organisms [10]. Analysis across several rivers and streams in North America report FLX levels between 13 and 540 ng/L [14], [15], well below the LC_50_ for the majority of aquatic species [16]. As FLX was the first SSRI marketed, it has the most abundant aquatic toxicological data, although this data set lacks the assessment of impacts on miRNAs and target gene responses.

The therapeutic target of FLX is the inhibition of the presynaptic membrane serotonin transporter (slc6a4), thereby inhibiting re-uptake of serotonin [17]. Importantly, serotonin is a key factor in satiety signaling and, as a side effect, patients taking Prozac exhibit weight loss such that some researchers consider Prozac as a potential therapy for obesity [18], [19]. In fish, feeding rates and the ability to capture prey significantly declined in fathead minnow and hybrid striped bass, respectively, to waterborne FLX doses ranging from 3.7 to ∼25 **μ**g/L [20]–[21]. The decline in feeding may be related to an apparent increase in expression of the corticotropin-releasing factor (CRF), a key food intake inhibitor in the hypothalamus. Mennigen et al. [22] demonstrated by repeated FLX injections in goldfish (*Carassius auratu*s) both decreased food intake and decreased body mass gain. Interestingly, the slc6a4 has also been identified in the liver of goldfish, among other tissues [23], suggesting there may be alternate target organs and pathways involved in the impacts of FLX exposure. Furthermore, Mennigen et al. [24] demonstrated that a chronic, 28-day environmentally relevant FLX exposure in goldfish impacted glucose metabolism, which was correlated with decreased hepatic gluconeogenesis. Although this study related the decline in feeding to FLX central disruption of anorexigenic peptides, it failed to address whether FLX has a direct impact on liver metabolism by acting through the slc6a4 transporter. Recently, Craig and Moon [25] examined the potential of zebrafish as a model for understanding energetics and fuel usage; they identified key transcriptomic regulators of liver metabolism related to fasting, particularly the master cellular energy sensor, adenosine monophosphate-activated protein kinase (AMPK). However, the upstream regulators of these pathways are poorly understood, although evidence from studies of mammalian models supports the inhibition of AMPK activity by serotonin, linking serotonergic pathways with energy metabolism [26]. Furthermore, there is evidence that miRNAs may also play a role regulating AMPK activity in dehydration-mediated metabolic depression in *Xenopus laevis* [27]. None of these studies examined a link between inhibition of serotonergic pathways and altered miRNA abundance.

The primary hypothesis of this study is that the miRNA profile of a zebrafish exposed to FLX will mimic that of a fasting response. Therefore, we examined the liver miRNA profiles of zebrafish that were either fed or fasted for 7 days, and fed or fasted and exposed to 540 ng/L FLX for the same period of time. The significantly expressed miRNA, as determined using a custom generated microarray, were subjected to *in silico* analysis of target genes and downstream pathways that could potentially be affected by the differentially expressed miRNAs. Results from this microarray study would then be subjected to target gene and pathway analyses, which would be used to compare the similarities between fasting and FLX exposed fish. These results when viewed with other FLX exposure studies will provide a greater understanding of potential mechanisms of endocrine disruption in aquatic species.

## Methods and Materials

### Animals and Experimental Design

Adult male and female zebrafish (*Danio rerio*) were purchased from Big Al’s Aquarium Services (Ottawa, ON) and maintained in 10-L acrylic tanks (n = 30 per tank) in closed, multi-rack aquatic housing systems (Aquatic Habitats, Apopka, FL). Fish were presumed to be of similar age based on size. All tanks were supplied with well-aerated dechloraminated City of Ottawa tap water at 28°C. Fish were maintained under a 12∶12-h light-dark cycle and fed once daily to satiation (food composition: crude protein 55%, crude fat 15%, crude fiber 1.5%, moisture 12%; Adult zebrafish complete diet, Zeigler Bros., Inc., Gardners, PA). Experimental procedures were approved by the University of Ottawa Animal Care Protocol Review Committee and undertaken in accordance with institutional animal care guidelines adhering to those of the Canadian Council on Animal Care. Zebrafish were sorted for size, sexed and weighed (0.496±0.02 g, n = 80). Only females were used for this experiment to avoid confounding of potential sex differences. Twenty fish were placed into 4 separate 8-L glass aquarium, provided with adequate aeration, and fish were exposed to either normal fish water or water that contained FLX at 540 ng/L (Sigma-Aldrich, Oakville, ON, Canada), a dose that reflects the highest concentration of FLX reported in the aquatic environment [15]. The experiment was designed as static renewal, where 80% of the water was replaced daily 1 h after feeding. Ammonia, nitrate and nitrites where measured and no differences were found between treatments (data not shown). An additional treatment of feeding and fasting was overlaid with the FLX exposure, yielding 4 treatment groups: Fed (which acts as the control), Fasted, Fed+FLX, and Fasted+FLX. An initial pilot study conducted revealed the greatest response of miRNA to fasting occurred after 7 days, and therefore the experiment was terminated at the end of the 7^th^ day. At the end of the exposure, zebrafish were terminally anesthetized with an overdose of 3-aminobenzoic acid ethylester (∼200 mg/L MS-222, Aqua Life, Syndel Laboratories Ltd., Vancouver, BC, Canada), livers were removed and pooled (2 livers per tube), immediately frozen on dry ice and stored at −80°C until processed.

### Extraction and Analysis of microRNA

Both total RNA and miRNA were extracted from frozen liver samples using the Absolutely RNA miRNA Purification Kit (Agilent Technologies, Santa Clara, CA, USA) following the manufacturer’s instructions. Purity and integrity of the extracted RNA was validated on a 2100 Bioanalyzer Instrument (Agilent Technologies), and only samples having a RNA Integrity Number >8.0 were used for further analysis. Four samples from each treatment were used for analysis of miRNA expression using a custom designed zebrafish miRNA 8×15K microarray (Agilent Technologies; G4474A). The design was based on the latest zebrafish miRNA database (miRbase) available as of September 2012 (270 distinct *Danio rerio* miRNA probes; www.mirbase.org) uploaded to Agilent’s proprietary custom design microarray software (eArray, Agilent Technologies). One particular highlight of the design is the 60 replications of each miRNA probe, which reduced overall hybridization noise and variability between samples. A total of 500 ng of total RNA (containing miRNA) was labeled and hybridized to the microarray following the explicit instructions of the miRNA Microarray System with miRNA Complete Labeling and Hyb Kit Protocol (Agilent Technologies). All reagents and kits used for labeling (i.e. Cyanine3-pCp), hybridization, and washes were purchased from Agilent Technologies. After hybridization and washing, the slides were immediately scanned on a SureScan Microarray Scanner G2600D (Agilent Technologies). Raw fluorescent data files were converted to normalized expression indices using the RMAalgorithm in GeneSpring GX (Agilent Technologies). Differential expression analysis used a one-way ANOVA with Tukey’s post-hoc test, and miRNA which demonstrated a 2.0-fold or higher change in expression between treatments were selected for further analysis. Predicted target gene analysis was performed using GeneSpring software linked to the zebrafish TargetScanFish database release 6.2 (www.targetscan.org/fish_62/;) [28]. This analysis created a predicted gene list that was further subjected to pathway analysis in GeneSpring GX, which provided an indication of targeted physiological/metabolic pathways. The results of this microarray experiment and all associated files were submitted to the Gene Expression Omnibus in accordance with MIAME guidelines (Accession number: GSE55229; http://www.ncbi.nlm.nih.gov/geo/).

### Microarray and Gene Target Validation

The main objective of this study was to examine the similarities that exist between FLX exposure and fasting, so the significantly regulated miRNAs that were common across the treatments compared to the control (fed) were examined. Using the same total RNA samples used to extract miRNAs, we validated the expression profile of the up-regulated miRNAs using real-time qPCR. Briefly, 1 **μ**g total RNA (containing miRNA) was reverse transcribed to miRNA 1^st^ strand cDNA using a kit purchased from Agilent Technologies (miRNA 1^st^ strand synthesis kit, Cat#600036). After cDNA synthesis, real-time qPCR was carried out using the Rotor-Gene Q real-time PCR thermocycler (Qiagen) and Rotor-Gene SYBR green PCR kit (Qiagen). Each reaction contained 5 **μ**l Rotor-Gene SYBR green PCR master mix (Qiagen), 1 **μ**l of each forward and reverse gene specific primers for a final concentration of 1 **μ**M, 2 **μ**l RNase/DNase-free purified H_2_O (Roche), and 1 **μ**l cDNA product described above. Cycling conditions were: 5 min initial denaturation at 95°C, 40 cycles of 95°C for 5 s, and 60°C for 10 s. Additionally, for validation that only one product was amplified, a melt curve analysis was used at the end of each run. To account for differences in amplification efficiencies between different cDNAs, standard curves were constructed for each target gene using serial dilutions of a reference pool of representative cDNA from all experiments. To account for differences in cDNA production and loading differences, all samples were normalized to the expression level of the housekeeping gene U11 snRNA [29], which did not change over the experimental treatments. Transcript expression data were calculated using the 2^ΔΔ–Ct^ method [30]. Both RNase/DNase-free H_2_O and non-reverse transcribed RNA were assayed on each plate to ensure no contamination was present in the reagents or in the primers used.

Target gene analysis revealed an extensive number of transcripts can be targeted by a single miRNA (see supplemental tables in File S1), however further exploration into these lists revealed that dre-let-7d and dre-miR-140-5p can specifically target the AMPK catalytic subunit 1 and 2, respectively. Primers were designed for AMPK**α**1, AMPK**α**2, slc6a4a and slc6a4b, and due to the similarity that exists between the isoforms, probe based PrimeTime Mini qPCR Assays (Integrated DNA Technologies, Coralville, IW, USA) were employed to decipher the expression differences between the two isoforms. Additional confirmation of target analysis was performed on two other transcripts that were *in silico* predicted targets: pyruvate carboxylase (pc), which maintains a regulatory role in gluconeogenesis, and the liver X receptor (nr1h3), which is involved in lipid and cholesterol metabolism (Tables S1–S5 in File S1). Probe-based and SYBR green qPCR assays were run on the Qiagen Rotor-Gene Q PCR machine as above, following the manufacturer’s instructions. Melt curves, standard curves and calculation of changes in expression were conducted as described above, with the exception that the housekeeping gene used was elongation factor 1 alpha (ef1**α**). All primers and probes for both miRNA and gene expression are found in Table 1.

**Table 1:** miRNA and gene primers used for validation of microarray experiment and confirmation of target gene prediction.

miRNA	miRBase #	Sequence
dre-let-7d	MIMAT0001762	TGAGGTAGTTGGTTGTATGGTT
dre-miR-140-5p	MIMAT0001836	CAGTGGTTTTACCCTATGGTAG
dre-miR-210-5p	MIMAT0003392	AGCCACTGACTAACGCACATTG
dre-miR-22b	MIMAT0001789	AAGCTGCCAGTTGAAGAGCTGT
dre-miR-301a	MIMAT0001870	CAGTGCAATAGTATTGTCAAAG
dre-miR-457b	MIMAT0001884	AAGCAGCACATAAATACTGGAG
**Gene**	**Accession #**	**Primers/Probe**
AMPK**α**1	NM_001110286	F: CAGTAATCCACCCCTGAGATG
		R: AGTTATCAGCACACCGACAG
		Probe: 56-FAM/TTGGATGAG/ZEN/AAGGAGAGCAGGCG/3IABkFQ
AMPK**α**2	XM_695739	F: GAACAGGTAGCCAGGAAGATC
		R: CTACATCCCCGAATACCTCAAC
		Probe: 56-FAM/TGCAGCATG/ZEN/AGCATCAGCAGACT/3IABkFQ
ef1**α**	NM_131263	F: GGAAATTCGAGACCAGCAAATAC
		R: TCAAACACTTCCACCTTCTCC
		Probe: 56-FAM/CACCACCAG/ZEN/CAACAATCAGCACA/3IABkFQ
nr1h3	NM_001017545	F: AGACAGCTCGTACCTCTACAG
		R: CTTTGGGCCGATCAATGAAAG
pc	NM_131550	F: GTACACCAACCTACACTTCCAG
		R: GATCGCCCACAATCTTAGAGG
slc6a4a	NM_001039972	F: GAGAATGGTTTGTGCTTGGC
		R:AGGAAGACAACGGTGATAACAG
		Probe: 56-FAM/TCCGTAGGT/ZEN/TAGAGTGGAGAGGGC/3IABkFQ
slc6a4b	NM_001177459	F: CAGATTCAGCAACGACATCAAG
		R: TCGAATAGCGTCAGGGTTTG
		Probe: 56-FAM/AGTCCTGGA/ZEN/GTGTAGCCCAACATC/3IABkFQ
U11 snRNA	NR_037952	F: GGGATTCTCTGACACAGTTACG
		R: CAAGACCAACGATCATTAGCAG

For gene expression analysis of AMPKα1 and 2 and slc6a4a and b isoforms, probe-based technologies was employed due to the high similarity between the 2 isoforms.

### Statistical Analysis

A statistical analysis of microarray data was performed as outlined above using the GeneSpring GX Software package v12.6 (Agilent). Linear and non-linear regression analyses for microarray validation and AMPK**α** isoform/miRNA relationship, respectively, were carried out using SigmaPlot Software v11.0 (Systat Software Inc., San Jose, CA, USA). Student’s t-test (SigmaPlot) was employed to determine the statistical significance between the relative abundance of the slc6a4 isoforms within each treatment. A one-way ANOVA and Tukey’s post-hoc test was employed for determining significant differences amongst the relative transcript abundance of pc and nr1h3 (p<0.05).

## Results

**Figure 1:**
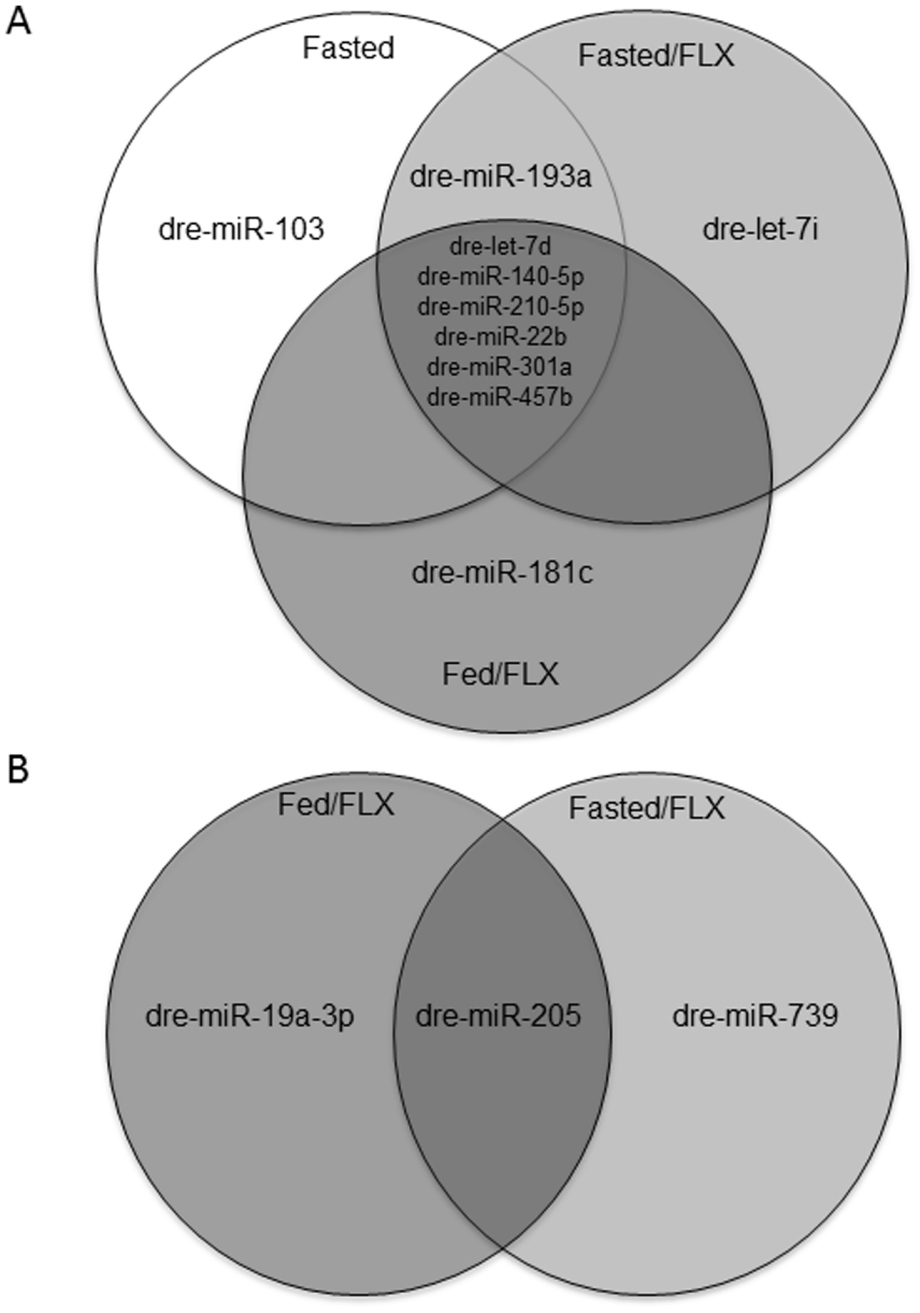
Venn diagrams of the overlapping expression patterns of miRNAs that were (A) up- and (B) down-regulated during fasting (Fasted), fasting with exposure to fluoxetine (540 ng/L; Fasted/FLX), and fed with exposure to fluoxetine (540 ng/L; Fed/FLX).

**Figure 2:**
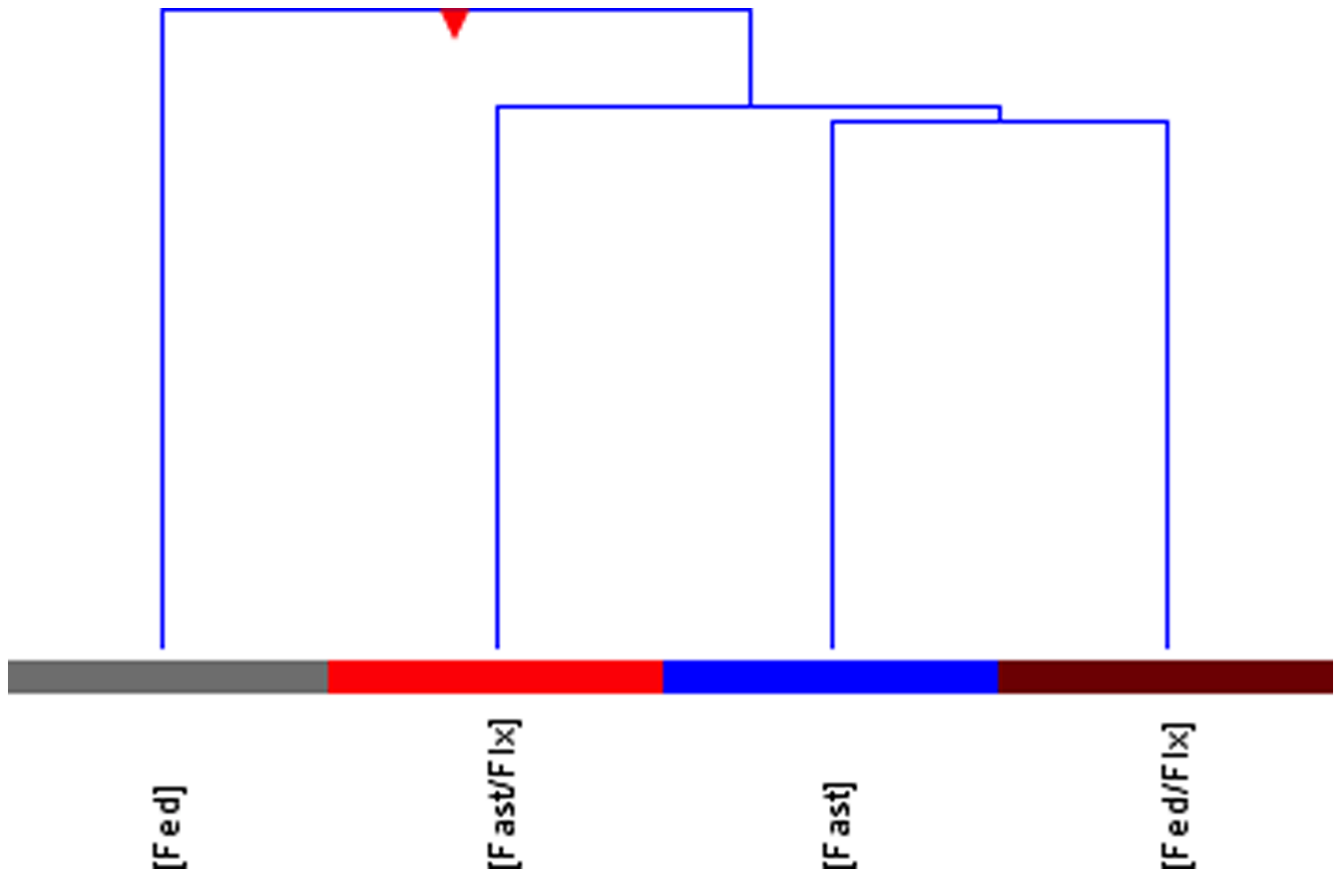
Cluster analysis demonstrating a significant relationship between individuals that were fasted and those exposed to fluoxetine versus those individuals who were only fed. [Fed]: Control; [Fast]: fasted only; [Fast/FLX]: fasted and exposed to 540 ng/L fluoxetine; [Fed/FLX]: fed and exposed to 540 ng/L fluoxetine.

**Figure 3:**
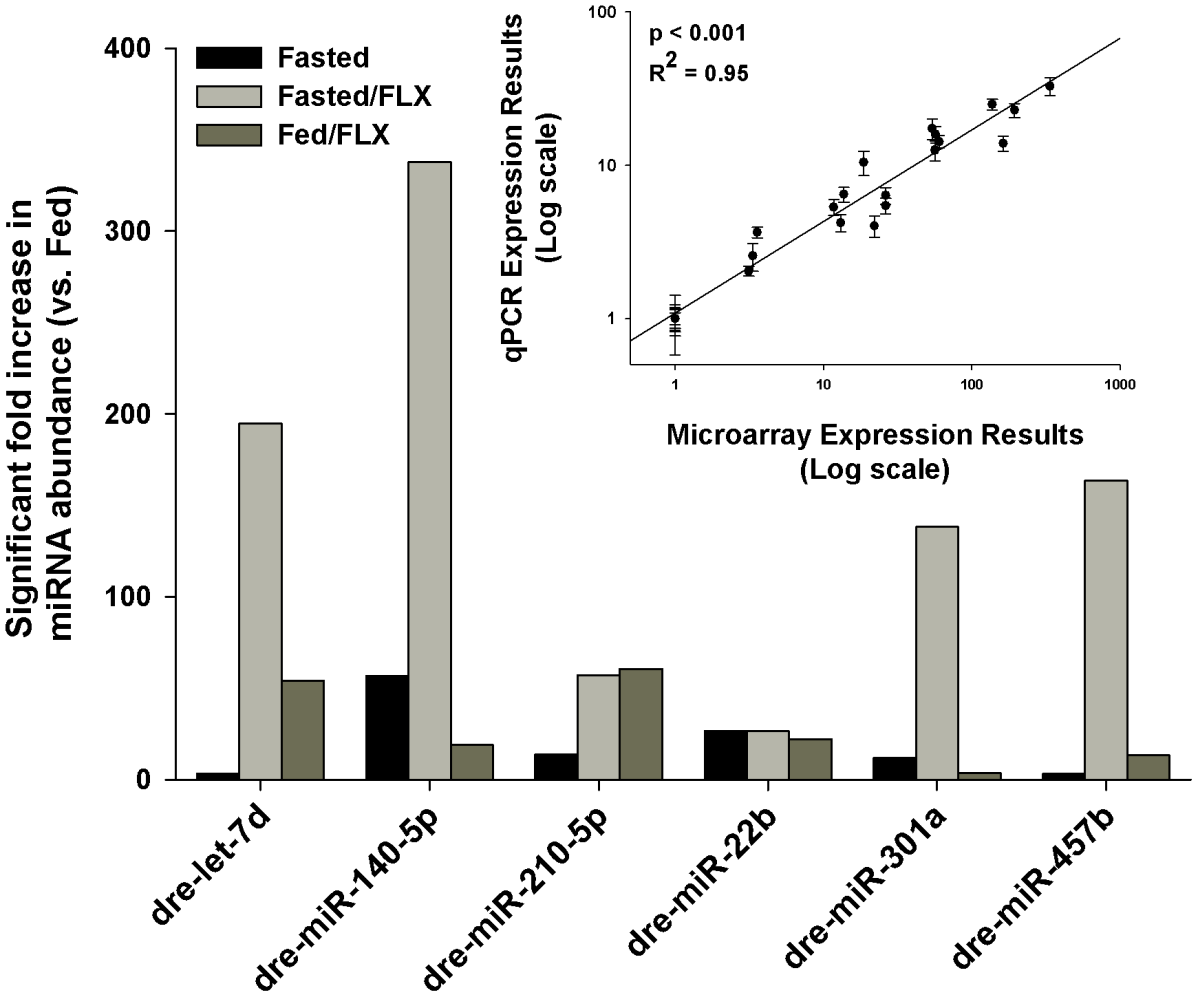
Abundance of the shared miRNAs that are significantly increased during fasting and fluoxetine exposure in the liver of zebrafish as determined by microarray experimentation. Inset is the validation of the significantly up-regulated miRNA by qPCR (R^2^ = 0.95, p<0.001).

A total of 13 of the 270 miRNAs significantly changed >2-fold in abundance for all experimental treatments compared with the fed control, representing 4.8% of the total population of miRNA targets represented on the microarray. Of these 13, 10 were significantly up-regulated, and only 3 were down-regulated (Figure 1A, B). Using hierarchical clustering analysis in the GeneSpring GX software package, there was a significant similarity found in the expression pattern of miRNA in zebrafish exposed to 7 days of fasting and those fed for 7 days in addition to being exposed to FLX at 540 ng/L (Figure 2). Furthermore, this clustering analysis revealed a greater similarity between all of the experimental treatments compared to the fed controls, supporting our hypothesis that FLX exposure mimics a fasting response (Figure 2). Comparison of the significantly up-regulated miRNAs revealed a similar, although variable, expression pattern of 6 specific miRNAs (Figure 3). What is apparent between treatments is how waterborne FLX exposure increased the magnitude of abundance of particular miRNAs. For example, dre-let-7d abundance increased nearly 200-fold when fasted zebrafish were exposed to FLX, and this pattern although smaller occurred for dre-miR-140-5p, dre-miR-301a, and dre-miR-457b (Figure 3). Overall, FLX exposure resulted in an increased expression profile of miRNAs compared with those of fasted zebrafish. To further support this finding, we used qPCR to validate the up-regulated miRNAs found on the microarray, and found a significant, strongly positive correlation between our qPCR results and the microarray expression pattern (R^2^ = 0.95, p<0.001; Figure 3 inset).

**Figure 4:**
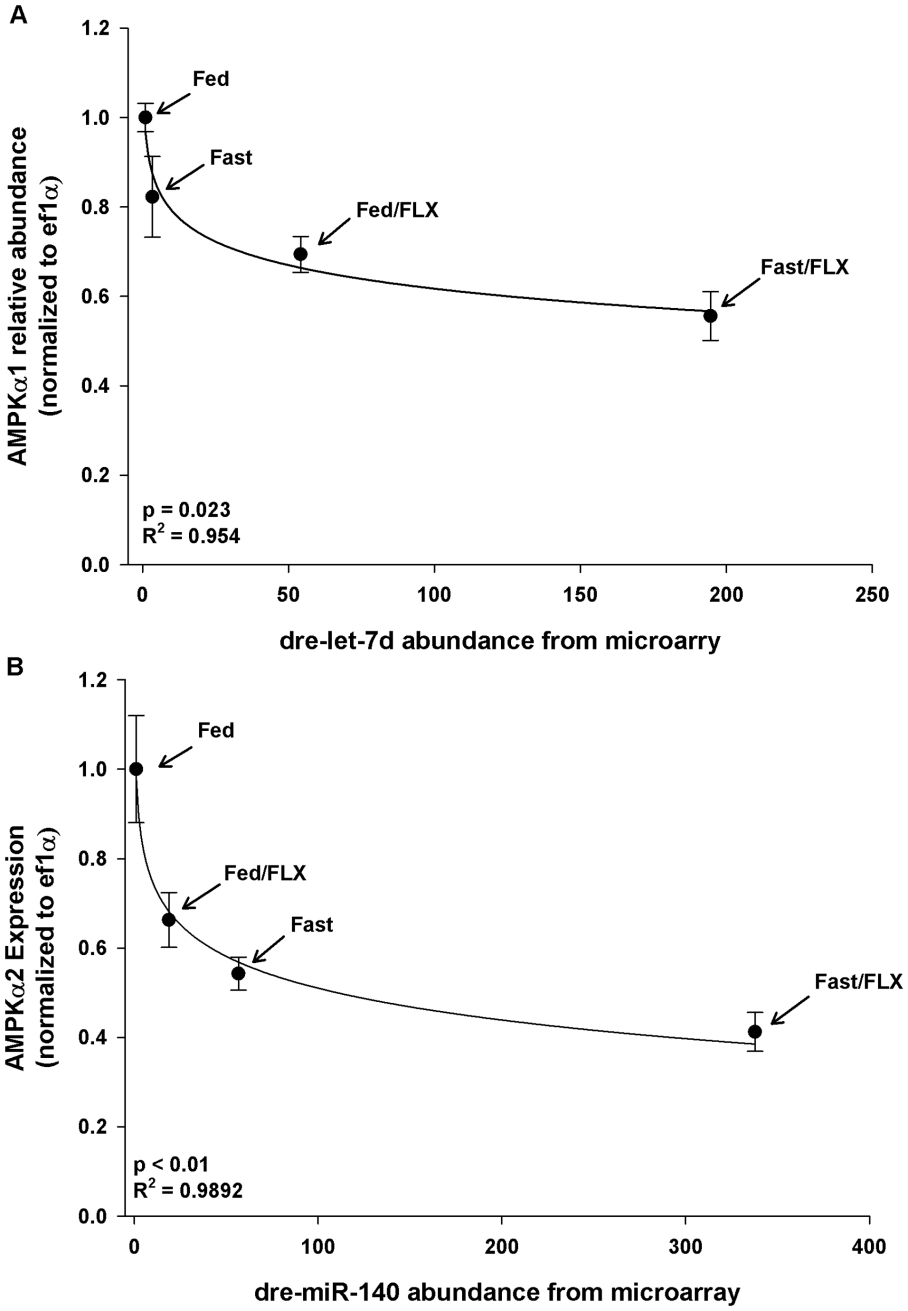
Relationship between miRNA abundance (from microarray experiment) of (A) dre-let-7d to AMPKα1, and (B) dre-miR-140a to AMPKα2. Values for AMPK relative transcript abundance are presented as the average ± S.E.M. (n = 4).

**Figure 5:**
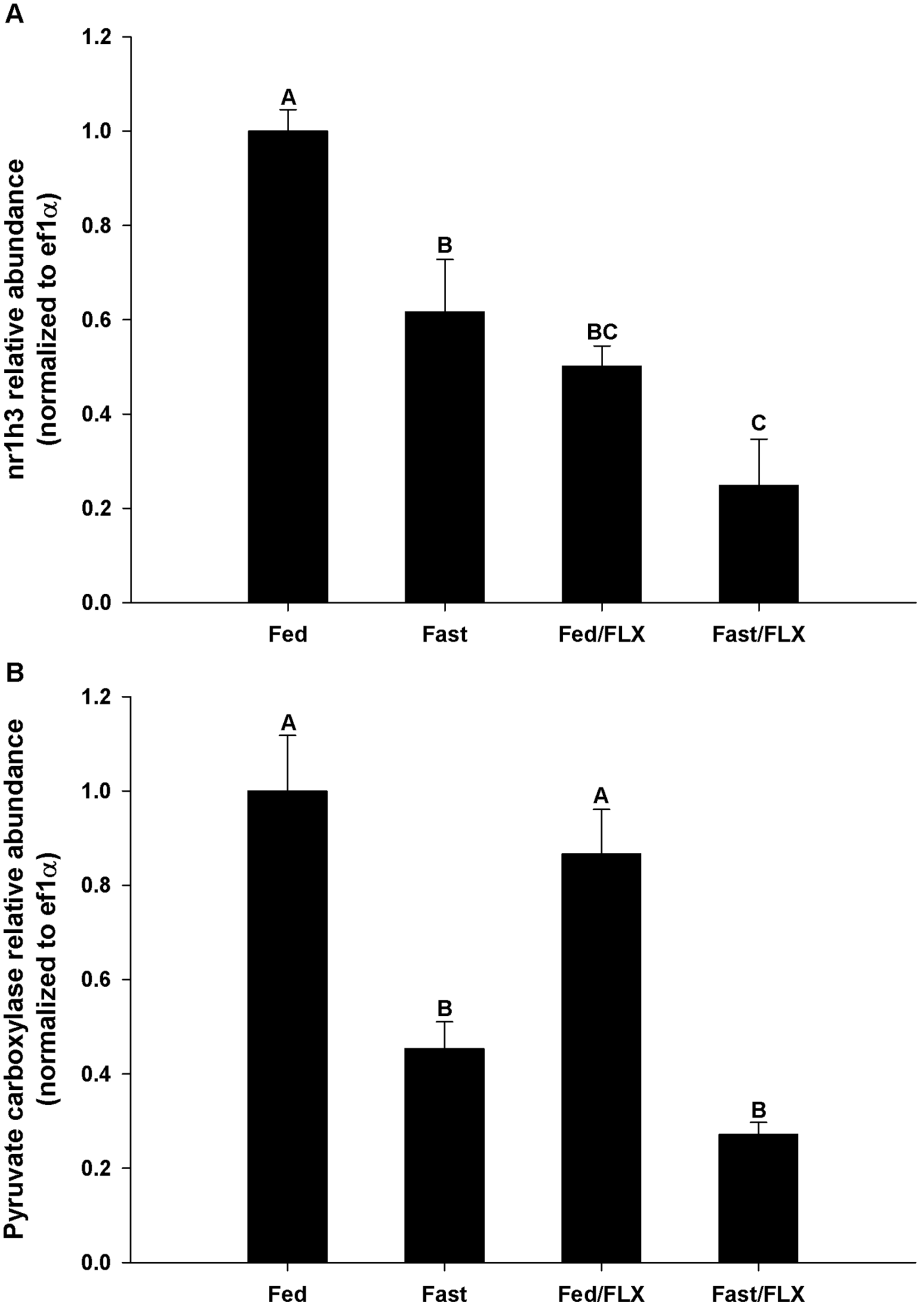
Relative transcript abundance of (A) nr1h3 (liver X receptor) and (B) pyruvate carboxylase to ef1α across all treatments. Values are presented as the average ± S.E.M. (n = 4). Bars that do not share a common letter are significantly different from each other, as determined by a one-way ANOVA and Tukey’s post-hoc test (p<0.05).

We further investigated the predicted gene targets that corresponded to each miRNA identified using the TargetScan Fish databases linked to the GeneSpring GX software. As miRNAs can bind to multiple targets, the gene targets list produced is exceptionally long (Tables S1-S5 in File S1). We discovered that two isoforms of AMPK, **α**1 and **α**2, which are the catalytic subunits of AMPK are regulated by two of the miRNAs found in our microarray experiment, dre-let-7d and dre-miR-140-5p, with a significant likelihood that these miRNAs could bind to the 3′UTR of their respective isoforms (Table 2). As miRNAs act to repress and/or degrade the expression of their gene target transcripts, we used qPCR to examine the profile of AMPK**α**1 and AMPK**α**2 mRNA expression in relation to their predicted miRNA ‘seed’ sequence. There was a significant, non-linear, relationship between the expressions of each isoform in relation to their specific miRNA (Figure 4A, B). Interestingly, the greater the likelihood that the miRNA will bind to its target, the greater the repression of the corresponding target. As dre-miR-140-5p had a higher probability of binding to AMPK**α**2 (p = 0.028 vs. p = 0.048; Tables S1-S3 in File S1), we observed a greater repression of transcript abundance when compared to AMPK**α**1 (∼60% vs. ∼40% repression). Furthermore, TargetScan analysis revealed that AMPK**α**2 has only 1 miRNA which is dre-miR-140-5p that is predicted to bind to the 3′UTR, whereas AMPK**α**1 has 19 potential miRNAs that can bind to the 3′UTR, including dre-let-7d. Additional examination of potential miRNA targets included qPCR validation of nr1h3 and pc (Figures 5A, B, respectively). There was a significant decrease in the relative abundance of nr1h3 across all treatments compared to the fed control, with a 50–75% decrease in association with FLX exposure (Figure 5A). Comparatively, there was ∼50 and ∼75% decrease in the relative transcript abundance of pyruvate carboxylase in the fasted and fasted/FLX exposures, respectively (Figure 5B). These results further confirmed the *in silico* prediction of specific miRNA targets associated with FLX exposure.

**Table 2:** The significantly up-regulated miRNA dre-let 7d and dre-miR-140a after fasting and fluoxetine exposure are predicted to target AMPK**α**1 and **α**2, respectively.

miRNA	Predicted target	Gene Symbol	p-value	Predicted pairing of target region	
dre-let-7d	AMPK**α**1	prkaa1	0.048	3′UTR =	5′…CUUUCUUCUAGAAUA**CUACCUC**U…
				miRNA =	3′ UUGGUAUGUUGGUU**GAUGGAG**U
dre-miR-140a	AMPK**α**2	prkaa2	0.028	3′UTR =	5′…GUCAUUGUGUUAAUU**AACCACU**U…
				miRNA =	3′ GAUGGUAUCCCAUU**UUGGUGA**C

Shown here are the predicted likelihoods that the miRNA will bind to it’s respective target and the ‘seed’ sequence relationship found in the 3′UTR of the target transcript.

By submitting the gene target list to Pathway Analysis in the GeneSpring software package, we identified potential pathways targeted by the miRNAs that were significantly down- or up-regulated. Importantly, it is known that miRNAs that are significantly up-regulated will repress their targeted transcripts [4], and therefore the pathways in which the targeted transcripts are involved would predictably be down-regulated. Conversely, down-regulated miRNA would be linked to up-regulated pathways. Using Pathway Analysis, a significant number of potentially down-regulated metabolic pathways were identified. These included adipogenesis, cholesterol biosynthesis, insulin signaling, lipid metabolism, and triacylglycerol synthesis (Table 3). Likely, a number of these anabolic pathways should be down-regulated under conditions of fasting for energy conservation, however these pathways are also down-regulated in zebrafish that were fed and exposed to FLX, supporting our hypothesis that FLX exposure mimics a fasting response. On the contrary, fewer pathways were found to be potentially up-regulated, likely due to the limited number of miRNA that are significantly repressed, although those pathways that are significantly linked to targeted transcripts were involved in stress and inflammation pathways including the p38 MAPK signaling and interleukin-6 signaling pathways (Table 3).

**Table 3:** The predicted up- and down-regulated pathways as determined from the subsequent list of target genes identified in the GeneSpring software package.

Predicted Down-regulatedPathway	WikipathwayID	p-value	MatchedGenes	Genes inPathway	% PathwayTargeted
Adipogenesis	WP1331	5.31E-12	6	98	6.1
Wnt Signalling	WP566	4.04E-10	5	185	2.7
G-Protein Signaling	WP1371	3.07E-08	4	75	5.3
Calcium Regulation inCardiac Cells	WP1365	3.07E-08	4	110	3.6
Myometrial Relaxation andContraction	WP1321	3.07E-08	4	110	3.6
EGFR1 Signaling	WP1323	3.07E-08	4	148	2.7
TNF-alpha NF-kB Signaling	WP1369	3.07E-08	4	153	2.6
Cholesterol Biosynthesis	WP1387	2.33E-06	3	14	21.4
Nuclear Receptors in Lipid Metabolisim	WP1326	2.33E-06	3	17	17.6
Triacylglyceride Synthesis	WP1347	2.33E-06	3	21	14.3
Nuclear Recpetos	WP1385	2.33E-06	3	28	10.7
SIDS Susceptibility	WP1377	2.33E-06	3	49	6.1
B-Cell Receptor Signaling	WP1354	2.33E-06	3	127	2.4
MAPK Signaling	WP1337	2.33E-06	3	131	2.3
Insulin Signaling	WP1313	2.33E-06	3	136	2.2
ERK1-ERK2 MAPK Cascade	WP402	2.33E-06	3	153	2.0
Biogenic Amine Synthesis	WP154	1.76E-04	2	11	18.2
Eicosanoid Synthesis	WP1318	1.76E-04	2	15	13.3
Ovarian Infertility Genes	WP1340	1.76E-04	2	22	9.1
Diurnally Regulated Genes	WP1379	1.76E-04	2	34	5.9
FAS Pathway & StressInduction of HSPs	WP511	1.76E-04	2	34	5.9
Selenium Metabolism	WP1358	1.76E-04	2	35	5.7
Circadian Exercise	WP562	1.76E-04	2	35	5.7
Apoptosis	WP1351	1.76E-04	2	60	3.3
Delta Norch Signaling	WP1382	1.76E-04	2	71	2.8
Cell Cycle	WP1393	1.76E-04	2	74	2.7
Nodal Signaling Pathway	WP341	1.76E-04	2	102	2.0
FGF Signaling	WP152	1.76E-04	2	131	1.5
**Predicted Up-regulated** **Pathway**	**Wikipathway** **ID**	**p-value**	**Matched** **Genes**	**Genes in** **Pathway**	**% Pathway** **Targeted**
p38 MAPK Signaling	WP1363	2.73E-09	4	30	13.3
TGF-beta Receptor Signaling	WP1367	2.73E-09	4	138	2.9
Id Signaling	WP1374	3.80E-07	3	47	6.4
G1 to S1 Cell Cycle Control	WP445	3.80E-07	3	48	6.3
IL-6 Signaling	WP1332	3.80E-07	3	88	3.4
Regulation of Actin Cytoskeleton	WP1380	3.80E-07	3	105	2.9
Kit Receptor Signaling	WP1341	5.27E-05	2	52	3.8
Toll-like Receptor Signaling	WP1384	5.27E-05	2	63	3.2
Wnt Signaling and Pluripotency	WP1344	5.27E-05	2	72	2.8

Tables are organized in descending order of the number of matched genes and likelihood to affect said pathway.

## Discussion

We hypothesized that the hepatic miRNA profile of a zebrafish exposed to FLX will mimic that of a fasting response. Cluster analysis of miRNA data indicated a strong relationship between fasting and the two treatments that involved FLX exposure compared with the fed group (Figure 2). Zebrafish have previously been established as a model for energetics, metabolism and fuel usage [25], and are a member of the Cyprinidae, the largest family of teleost fishes. This is important for our comparative approach in understanding the metabolic impacts of FLX. Here, we have identified 6 specific miRNAs that have a distinct overlap between fasting and FLX exposure conditions. The expression amplitudes of these miRNAs are increased when FLX is present (Figure 3). For example, dre-let-7d, dre-miR-140-5p, dre-miR-301a, and dre-miR-457b all increased in abundance >100-fold compared with the fasting only treatment. Functionally, miRNAs are effective at blocking target transcript translation by means of abundance [31], and therefore, with a high abundance of these miRNAs, target transcript repression is more likely to occur. However, this response appears to be muted when zebrafish were fed, although a significant increase in abundance versus fasting alone still persisted (Figure 3). Taking these miRNAs into account, we can predict *in silico* potential target genes and target pathways that would be either up- or down-regulated with respect to miRNA abundance. What is most apparent is that the changes in miRNA abundance are primarily directed at increased as opposed to decreased miRNAs suggesting that both fasting and FLX exposures resulted in down-regulation of a broad array of metabolic pathways. This is confirmed through pathway analysis indicating 28 down-regulated pathways are shared between FLX and fasting exposures, and only 9 pathways are up-regulated related to FLX exposures alone (Table 3). The majority of pathways down-regulated by FLX exposure and/or fasting are anabolic pathways, such as adipogenesis, cholesterol biosynthesis, and triacyglycerol synthesis, confirming some of the metabolic alterations Mennigen et al. [24] established in goldfish exposed to FLX. Further investigation into the pathway analysis included examining the relative abundance of key transcripts involved in some of these metabolic pathways. Across all experimental treatments, there was a significant decrease in the relative abundance of nr1h3 (Figure 5A), a nuclear receptor that is a key regulator of lipid and cholesterol metabolism and transport [32]. Pyruvate carboxylase, a key enzyme required for gluconeogenesis and supplying cytoplasmic NADPH for lipogenesis [33], [34], was significantly repressed during fasting and fasting+FLX exposure (Figure 5B). Combined, these qPCR validation experiments confirmed the target prediction and pathway analysis that fuel metabolism is significantly altered during FLX exposure, although future studies will tease apart these specific pathways and determine their functional consequence in relation to FLX exposure and energy metabolism. Pathways that are up-regulated, such as the p38 MAPK and interleukin signaling pathways, are likely attributed to the stress of FLX exposure, as these are key pathways involved in the stress and inflammation response [35], [36]. Having altered fuel capacity and increased inflammation during FLX exposure despite being fed, puts zebrafish exposed to waterborne FLX at a distinct metabolic disadvantage resulting in an anorectic state and perhaps a reduced overall fitness [37].

Fluoxetine is a prominent psychotropic drug prescribed for the treatment of depression and anxiety, and is a commonly detected pharmaceutical in wastewaters [15], [38]. Furthermore, there is evidence that FLX can bio-concentrate in tissues of fish exposed to waterborne FLX. Accumulation of FLX has been detected in the brain, liver, and skeletal muscles of numerous fish species in North America [39], [40]. Moreover, studies involving Japanese medaka have indicated that the accumulation of FLX occurs due to the inability of the fish to rapidly metabolize and excrete the drug, likely resulting in further non-specific damage as a result of increased tissue FLX concentrations [41], [42]. Knowing waterborne FLX can accumulate in tissues, Mennigen et al. [24] examined the metabolic consequences of FLX exposure in goldfish and determined there was a significant impact on liver glucose metabolism at 540 ng/L, and suggested that the link between the FLX exposure and liver impact was driven by the presence of the serotonergic transporter slc6a4. Here, we were able to identify and determine the relative abundance of two slc6a4 transporter transcripts (Figure S1 in File S1) in the liver of zebrafish, and although no changes in transcript abundance to either fasting or FLX exposure were noted, the presence of these transcripts implies that FLX can directly affect hepatic serotonin uptake. However, further studies are needed to establish the link between serotonergic signaling pathways, protein abundance of slc6a4, and the alteration in expression of various miRNA.

The paramount role of serotonin as a neurotransmitter and importance in numerous neurological disorders is well documented [43]. Numerous therapeutic drugs including FLX, are being widely prescribed to control depression. However, the role of serotonin in peripheral systems is less well-described. Approximately 90% of serotonin produced in mammalian systems comes from the gastrointestinal tract, and aside from the aforementioned role in neurotransmission, serotonin maintains peripheral roles in gastrointestinal motility, cell proliferation, vasculature contractility and relaxation, and apoptosis [44]. Most intriguing is the recently discovered effects serotonin exerts on the liver. In a mammalian model, platelet-derived serotonin mediates the signaling cascade involved in liver regeneration, which involves both the serotonin transporter and receptor found on the surface of hepatocytes, thereby initiating cellular proliferation [45]. Hence, the presence of scl6a4 in hepatocytes indicates that this transporter is a potential target for FLX, and by blocking it, liver metabolic dysfunction is likely, which is validated by the target pathway analysis. However, this does not rule out the possibility that centrally derived signals (other hormones and neuropeptides) could have contributed to the alter expression of liver miRNA. Although the Fed+FLX treatment group exhibited a pathway profile similar to that of a fasted zebrafish, these fish were visually confirmed to have food in their gut at the time of sacrifice. While Mennigan et al. [24] demonstrated goldfish had a reduced appetite following FLX exposure, it was apparent that zebrafish in this experiment did eat, although how much is unknown. As the target profile indicates, anabolic pathways were down-regulated with FLX exposure, suggesting that energy from food being ingested is not being efficiently assimilated into the necessary metabolites for usage. Inefficient energy assimilation can have direct impacts on egg production, fecundity, and overall fitness [46], which implies that exposure to FLX at environmentally-relevant concentrations will disrupt not only metabolism but may also impact species abundance. Indeed, FLX has been shown to reduce ovulation in zebrafish [47], and exposure to identical environmentally-relevant levels of FLX also blocks the ability of male goldfish to release sperm in response to female sex pheromones [23].

Fasting in zebrafish as in mammals, results in switching cellular metabolism from anabolic to catabolic pathways [25]. One of the master regulators of this switch is AMPK, which is a highly conserved kinase that functions as a master regulator of metabolism during states of low energy availability; AMPK becomes actively phosphorylated when cellular AMP levels are high, and this switch to catabolic pathways aids in maintaining the cellular ATP homeostasis [48]. Zebrafish increase the hepatic AMPK**α** catalytic subunit mRNA levels during prolonged fasting (3 weeks); however the transcriptional regulation of the protein was not examined [25]. Here we demonstrate that two of the significantly up-regulated miRNAs by all the experimental treatments in zebrafish target the two isoforms of AMPK**α** (Tables S1-S3 in File S1). Further examination using TargetScanFish revealed 37 different miRNA families have the predicted potential to regulate the expression of AMPK**α**1, whereas only one miRNA (dre-miR-140a) binds to the 3′UTR of AMPK**α**2. However, this change in transcript abundance does not predict either the protein abundance or AMPK activity. Nevertheless, this is the first time a link between miRNA and the master metabolic regulator AMPK has been established in a fish model, and future studies examining the metabolic disruption should take this into account.

In conclusion, this is the first characterization of the miRNA profile in relation to fasting and FLX exposure in zebrafish when compared to nominally fed zebrafish. We were able to confirm our hypothesis that the miRNA fingerprint of FLX exposure mimics that of a fasting response. The relevance of these observations supports the findings in related cyprinids and other teleosts [37], which points towards a metabolic disruption and reduced overall fitness of fish exposed to FLX. These results using FLX which is amongst a list of detectable SSRIs in waterways in North America [14], [15], further supports the need for proper regulation and remediation to prevent potential detrimental effects on fish in contaminated aquatic ecosystems.

## Supporting Information

File S1 (DOCX)
