# Supplementary material for: Profiling Hepatic microRNAs in Zebrafish: Fluoxetine Exposure Mimics a Fasting Response That Targets AMP-Activated Protein Kinase (AMPK)
Source: PLoS One. 2014 Apr 21;9(4):e95351. doi: 10.1371/journal.pone.0095351 (PMC3994061; doi:10.1371/journal.pone.0095351)

Table S1: Significantly (>2.0 fold) up-regulated miRNA in the liver of zebrafish fasted for 7 days and their *in silico* predicted target genes. Target genes are sorted by ascending level of significant likelihood of targeting said gene. Legend: MIMAT0001762 = dre-let-7d; MIMAT0001789 = dre-miR-22b; MIMAT0001816 = dre-miR-103; MIMAT0001836 = dre-miR-140-5p; MIMAT0001856 = dre-miR-193a; MIMAT0001870 = dre-miR-301a; MIMAT0001884 = dre-miR-457b.

|  | **mirbase accession No** | **p-value** | **GeneID** | **Symbol** | **Description** |
| --- | --- | --- | --- | --- | --- |
| 1 | MIMAT0001870,MIMAT0001836,MIMAT0001856,MIMAT0001852,MIMAT0001816,MIMAT0001884,MIMAT0001762 | 0.00005 | 497350 | im:7137555 | im:7137555 |
| 2 | MIMAT0001870,MIMAT0001884,MIMAT0001789,MIMAT0001856,MIMAT0001852,MIMAT0001816 | 0.00269 | 558048 | n4bp2 | NEDD4 binding protein 2 |
| 3 | MIMAT0001836,MIMAT0001852,MIMAT0001789 | 0.00496 | 322462 | sult6b1 | sulfotransferase family, cytosolic, 6b, member 1 |
| 4 | MIMAT0001789,MIMAT0001836,MIMAT0001852,MIMAT0001816 | 0.00638 | 555279 | tmem209 | transmembrane protein 209 |
| 5 | MIMAT0001836,MIMAT0001856,MIMAT0001852,MIMAT0001816,MIMAT0001884 | 0.00739 | 569099 | adamts6 | ADAM metallopeptidase with thrombospondin type 1 motif, 6 |
| 6 | MIMAT0001870,MIMAT0001836,MIMAT0001852,MIMAT0001789 | 0.00868 | 30537 | emx2 | empty spiracles homeobox 2 |
| 7 | MIMAT0001856,MIMAT0001852,MIMAT0001816,MIMAT0001884 | 0.00868 | 336606 | hadhb | hydroxyacyl-Coenzyme A dehydrogenase |
| 8 | MIMAT0001870,MIMAT0001856,MIMAT0001816,MIMAT0001884 | 0.00868 | 406271 | etfb | electron-transfer-flavoprotein, beta polypeptide |
| 9 | MIMAT0001836,MIMAT0001856,MIMAT0001816,MIMAT0001884 | 0.00868 | 562912 | ltbp3 | latent transforming growth factor beta binding protein 3 |
| 10 | MIMAT0001870,MIMAT0001884,MIMAT0001789,MIMAT0001852,MIMAT0001816,MIMAT0001762 | 0.00979 | 368893 | acin1a | apoptotic chromatin condensation inducer 1a |
| 11 | MIMAT0001836,MIMAT0001856,MIMAT0001852,MIMAT0001816 | 0.01002 | 565922 | ift46 | intraflagellar transport protein 46 |
| 12 | MIMAT0001836,MIMAT0001852,MIMAT0001816 | 0.01036 | 791844 | ca15b | carbonic anhydrase XV b |
| 13 | MIMAT0001836,MIMAT0001856,MIMAT0001852,MIMAT0001816,MIMAT0001884 | 0.01140 | 317747 | rock2a | rho-associated, coiled-coil containing protein kinase 2a |
| 14 | MIMAT0001836,MIMAT0001856,MIMAT0001852,MIMAT0001816,MIMAT0001884 | 0.01261 | 557556 | dlgap3 | discs, large (Drosophila) homolog-associated protein 3 |
| 15 | MIMAT0001870,MIMAT0001836,MIMAT0001852,MIMAT0001816,MIMAT0001762 | 0.01261 | 563087 | si:ch211-129c21.1 | si:ch211-129c21.1 |
| 16 | MIMAT0001852,MIMAT0001816,MIMAT0001884,MIMAT0001762,MIMAT0001789 | 0.01261 | 569471 | chd7 | chromodomain helicase DNA binding protein 7 |
| 17 | MIMAT0001870,MIMAT0001836,MIMAT0001884,MIMAT0001856,MIMAT0001852,MIMAT0001816 | 0.01261 | 556544 | si:dkey-199f5.8 | si:dkey-199f5.8 |
| 18 | MIMAT0001870,MIMAT0001836,MIMAT0001816,MIMAT0001884 | 0.01312 | 79377 | gfra1b | gdnf family receptor alpha 1b |
| 19 | MIMAT0001870,MIMAT0001884,MIMAT0001856,MIMAT0001852,MIMAT0001816,MIMAT0001762 | 0.01368 | 322903 | wu:fb76c12 | wu:fb76c12 |
| 20 | MIMAT0001856,MIMAT0001852,MIMAT0001816,MIMAT0001884,MIMAT0001762 | 0.01391 | 100526672 | ptprsb | protein tyrosine phosphatase, receptor type, s, b |
| 21 | MIMAT0001870,MIMAT0001836,MIMAT0001852,MIMAT0001816,MIMAT0001884 | 0.01391 | 569727 | phip | pleckstrin homology domain interacting protein |
| 22 | MIMAT0001870,MIMAT0001852,MIMAT0001816,MIMAT0001884,MIMAT0001789 | 0.01530 | 393187 | atp5s | ATP synthase, H+ transporting, mitochondrial F0 complex, subunit s |
| 23 | MIMAT0001870,MIMAT0001836,MIMAT0001856,MIMAT0001884,MIMAT0001789 | 0.01530 | 541316 | lrrc1 | leucine rich repeat containing 1 |
| 24 | MIMAT0001836,MIMAT0001884,MIMAT0001789,MIMAT0001852,MIMAT0001816 | 0.01680 | 553226 | tmem198a | transmembrane protein 198a |
| 25 | MIMAT0001836,MIMAT0001852,MIMAT0001816,MIMAT0001884 | 0.01681 | 557269 | pdzd3a | PDZ domain containing 3a |
| 26 | MIMAT0001836,MIMAT0001852,MIMAT0001816,MIMAT0001884 | 0.01681 | 58120 | nme6 | non-metastatic cells 6, protein expressed in (nucleoside-diphosphate kinase) |
| 27 | MIMAT0001870,MIMAT0001856,MIMAT0001816,MIMAT0001789 | 0.01681 | 677750 | myl2b | myosin, light polypeptide 2b, regulatory, cardiac, slow |
| 28 | MIMAT0001836,MIMAT0001852,MIMAT0001816 | 0.01832 | 445307 | dexi | dexamethasone-induced transcript |
| 29 | MIMAT0001884,MIMAT0001852,MIMAT0001816 | 0.01832 | 768151 | dennd2da | DENN/MADD domain containing 2Da |
| 30 | MIMAT0001836,MIMAT0001852,MIMAT0001816,MIMAT0001884 | 0.01889 | 406720 | golga5 | golgi autoantigen, golgin subfamily a, 5 |
| 31 | MIMAT0001836,MIMAT0001852,MIMAT0001816,MIMAT0001884 | 0.02113 | 557906 | spsb4a | splA/ryanodine receptor domain and SOCS box containing 4a |
| 32 | MIMAT0001884,MIMAT0001852,MIMAT0001816 | 0.02157 | 323005 | nktr | natural killer-tumor recognition sequence |
| 33 | MIMAT0001884,MIMAT0001852,MIMAT0001816 | 0.02157 | 436793 | cbln1 | cerebellin 1 precursor |
| 34 | MIMAT0001884,MIMAT0001816 | 0.02345 | 386640 | cep97 | centrosomal protein 97 |
| 35 | MIMAT0001856,MIMAT0001789 | 0.02345 | 562337 | si:dkeyp-53d3.3 | si:dkeyp-53d3.3 |
| 36 | MIMAT0001856,MIMAT0001816 | 0.02345 | 563506 | tiparp | TCDD-inducible poly(ADP-ribose) polymerase |
| 37 | MIMAT0001852,MIMAT0001816,MIMAT0001884,MIMAT0001762 | 0.02355 | 692316 | atxn7l2a | ataxin 7-like 2a |
| 38 | MIMAT0001870,MIMAT0001836,MIMAT0001789,MIMAT0001856,MIMAT0001852,MIMAT0001762 | 0.02498 | 565854 | onecut3 | one cut homeobox 3 |
| 39 | MIMAT0001884,MIMAT0001852,MIMAT0001816 | 0.02515 | 100007702 | rspo3 | R-spondin 3 homolog (Xenopus laevis) |
| 40 | MIMAT0001789,MIMAT0001836,MIMAT0001852 | 0.02515 | 405810 | ehd1a | EH-domain containing 1a |
| 41 | MIMAT0001836 | 0.02804 | 572074 | prkaa2 | protein kinase, AMP-activated, alpha 2 catalytic subunit |
| 42 | MIMAT0001836,MIMAT0001789,MIMAT0001852,MIMAT0001816,MIMAT0001762 | 0.02809 | 30771 | hic1l | hypermethylated in cancer 1 like |
| 43 | MIMAT0001870,MIMAT0001836,MIMAT0001884,MIMAT0001852,MIMAT0001816 | 0.02809 | 570147 | add2 | adducin 2 (beta) |
| 44 | MIMAT0001836,MIMAT0001816,MIMAT0001884,MIMAT0001762 | 0.02891 | 436928 | atxn7l2b | ataxin 7-like 2b |
| 45 | MIMAT0001836,MIMAT0001856,MIMAT0001816 | 0.02904 | 494106 | nsun4 | NOL1/NOP2/Sun domain family, member 4 |
| 46 | MIMAT0001836,MIMAT0001884,MIMAT0001852,MIMAT0001816,MIMAT0001762 | 0.03039 | 548341 | nr1h3 | nuclear receptor subfamily 1, group H, member 3 |
| 47 | MIMAT0001870,MIMAT0001836,MIMAT0001884,MIMAT0001856,MIMAT0001816 | 0.03039 | 557456 | trpc5a | transient receptor potential cation channel, subfamily C, member 5a |
| 48 | MIMAT0001852,MIMAT0001816 | 0.03067 | 393398 | ctssa | cathepsin S, a |
| 49 | MIMAT0001836,MIMAT0001856,MIMAT0001884,MIMAT0001789 | 0.03186 | 100005490 | zrsr2 | zinc finger (CCCH type), RNA-binding motif and serine/arginine rich 2 |
| 50 | MIMAT0001836,MIMAT0001852,MIMAT0001816,MIMAT0001884 | 0.03186 | 324180 | txlna | taxilin alpha |
| 51 | MIMAT0001870,MIMAT0001816,MIMAT0001884,MIMAT0001762 | 0.03186 | 405787 | fibpb | fibroblast growth factor (acidic) intracellular binding protein b |
| 52 | MIMAT0001836,MIMAT0001852,MIMAT0001816,MIMAT0001884 | 0.03186 | 557482 | rab3b | RAB3B, member RAS oncogene family |
| 53 | MIMAT0001870,MIMAT0001856,MIMAT0001884,MIMAT0001789 | 0.03186 | 562525 | cldn2 | claudin 2 |
| 54 | MIMAT0001870,MIMAT0001836,MIMAT0001816,MIMAT0001789 | 0.03186 | 566591 | furinb | furin (paired basic amino acid cleaving enzyme) b |
| 55 | MIMAT0001789,MIMAT0001836,MIMAT0001852 | 0.03325 | 325469 | fbxo9 | F-box protein 9 |
| 56 | MIMAT0001884,MIMAT0001852,MIMAT0001816 | 0.03325 | 393757 | chchd10 | coiled-coil-helix-coiled-coil-helix domain containing 10 |
| 57 | MIMAT0001884,MIMAT0001852,MIMAT0001816 | 0.03325 | 555430 | tmie | transmembrane inner ear |
| 58 | MIMAT0001884,MIMAT0001852,MIMAT0001816 | 0.03325 | 555825 | sb:cb54 | sb:cb54 |
| 59 | MIMAT0001870,MIMAT0001836,MIMAT0001884,MIMAT0001856,MIMAT0001852,MIMAT0001816 | 0.03499 | 798995 | tbc1d23 | TBC1 domain family, member 23 |
| 60 | MIMAT0001870,MIMAT0001856,MIMAT0001852,MIMAT0001789 | 0.03500 | 114432 | btg1 | B-cell translocation gene 1 |
| 61 | MIMAT0001856,MIMAT0001852,MIMAT0001816,MIMAT0001884 | 0.03500 | 245697 | flot1b | flotillin 1b |
| 62 | MIMAT0001870,MIMAT0001836,MIMAT0001816,MIMAT0001884 | 0.03500 | 393293 | hsd11b3a | hydroxysteroid (11-beta) dehydrogenase 3a |
| 63 | MIMAT0001870,MIMAT0001816,MIMAT0001884,MIMAT0001789 | 0.03500 | 570808 | pggt1b | protein geranylgeranyltransferase type I, beta subunit |
| 64 | MIMAT0001856,MIMAT0001852,MIMAT0001816,MIMAT0001884 | 0.03500 | 58068 | pc | pyruvate carboxylase |
| 65 | MIMAT0001870,MIMAT0001884,MIMAT0001789,MIMAT0001852,MIMAT0001816 | 0.03539 | 558427 | thrab | thyroid hormone receptor alpha b |
| 66 | MIMAT0001836 | 0.03738 | 678590 | zgc:136254 | zgc:136254 |
| 67 | MIMAT0001856,MIMAT0001852,MIMAT0001816 | 0.03778 | 368326 | il13ra2 | interleukin 13 receptor, alpha 2 |
| 68 | MIMAT0001836,MIMAT0001856,MIMAT0001816 | 0.03778 | 556710 | taf10 | TAF10 RNA polymerase II, TATA box binding protein (TBP)-associated factor |
| 69 | MIMAT0001836,MIMAT0001852,MIMAT0001816 | 0.03778 | 569550 | edn2 | endothelin 2 |
| 70 | MIMAT0001836,MIMAT0001852,MIMAT0001816,MIMAT0001884 | 0.03833 | 100126070 | cables1 | Cdk5 and Abl enzyme substrate 1 |
| 71 | MIMAT0001856,MIMAT0001852,MIMAT0001816,MIMAT0001762 | 0.03833 | 30238 | eng2b | engrailed 2b |
| 72 | MIMAT0001836,MIMAT0001856,MIMAT0001816,MIMAT0001884 | 0.03833 | 393236 | sybl1 | synaptobrevin-like 1 |
| 73 | MIMAT0001870,MIMAT0001836,MIMAT0001816,MIMAT0001884 | 0.03833 | 560560 | ywhag2 | 3-monooxygenase/tryptophan 5-monooxygenase activation protein, gamma polypeptide 2 |
| 74 | MIMAT0001856,MIMAT0001852,MIMAT0001816,MIMAT0001789 | 0.03833 | 564337 | ppp3r1a | protein phosphatase 3 (formerly 2B), regulatory s1ubunit B, alpha isoform, a |
| 75 | MIMAT0001852,MIMAT0001816,MIMAT0001884,MIMAT0001789 | 0.03833 | 567119 | fem1b | fem-1 homolog b (C. elegans) |
| 76 | MIMAT0001836,MIMAT0001852,MIMAT0001816,MIMAT0001762 | 0.03833 | 58090 | trpc4apa | transient receptor potential cation channel, subfamily C, member 4 associated protein a |
| 77 | MIMAT0001852,MIMAT0001816 | 0.03869 | 405848 | tmed7 | transmembrane emp24 protein transport domain containing 7 |
| 78 | MIMAT0001836,MIMAT0001856 | 0.03869 | 405880 | zgc:85843 | zgc:85843 |
| 79 | MIMAT0001870,MIMAT0001836,MIMAT0001884,MIMAT0001852,MIMAT0001816 | 0.04093 | 791150 | gripap1 | GRIP1 associated protein 1 |
| 80 | MIMAT0001870,MIMAT0001856,MIMAT0001852,MIMAT0001789 | 0.04186 | 559732 | ttc9b | tetratricopeptide repeat domain 9B |
| 81 | MIMAT0001870,MIMAT0001836,MIMAT0001884,MIMAT0001852,MIMAT0001816,MIMAT0001762 | 0.04228 | 260350 | has2 | hyaluronan synthase 2 |
| 82 | MIMAT0001870,MIMAT0001836,MIMAT0001884,MIMAT0001856,MIMAT0001852,MIMAT0001816 | 0.04228 | 563570 | hs3st4 | heparan sulfate (glucosamine) 3-O-sulfotransferase 4 |
| 83 | MIMAT0001884,MIMAT0001856,MIMAT0001816 | 0.04264 | 431723 | tha1 | threonine aldolase 1 |
| 84 | MIMAT0001884,MIMAT0001870,MIMAT0001852 | 0.04264 | 550376 | cib1 | calcium and integrin binding 1 (calmyrin) |
| 85 | MIMAT0001870,MIMAT0001789,MIMAT0001852 | 0.04264 | 558261 | ociad2 | OCIA domain containing 2 |
| 86 | MIMAT0001884,MIMAT0001836,MIMAT0001816 | 0.04264 | 561678 | si:ch211-260p9.6 | si:ch211-260p9.6 |
| 87 | MIMAT0001884,MIMAT0001852,MIMAT0001816 | 0.04264 | 563304 | ino80db | INO80 complex subunit Db |
| 88 | MIMAT0001870,MIMAT0001852,MIMAT0001816 | 0.04264 | 664741 | inpp4aa | inositol polyphosphate-4-phosphatase, type Ia |
| 89 | MIMAT0001836,MIMAT0001884,MIMAT0001789,MIMAT0001852,MIMAT0001816 | 0.04392 | 562839 | zgc:123349 | rad23ab |
| 90 | MIMAT0001836,MIMAT0001884,MIMAT0001852,MIMAT0001816,MIMAT0001762 | 0.04392 | 569093 | zgc:114104 | zgc:114104 |
| 91 | MIMAT0001870,MIMAT0001884,MIMAT0001789,MIMAT0001852,MIMAT0001816,MIMAT0001762 | 0.04495 | 550555 | slc35e4 | solute carrier family 35, member E4 |
| 92 | MIMAT0001852,MIMAT0001816,MIMAT0001884,MIMAT0001762 | 0.04559 | 565672 | rnpc3 | RNA-binding region (RNP1, RRM) containing 3 |
| 93 | MIMAT0001852,MIMAT0001816 | 0.04744 | 394041 | cbll1 | Cas-Br-M (murine) ecotropic retroviral transforming sequence-like 1 |
| 94 | MIMAT0001852,MIMAT0001789 | 0.04744 | 406266 | rsl24d1 | ribosomal L24 domain containing 1 |
| 95 | MIMAT0001836,MIMAT0001852 | 0.04744 | 450063 | mogat2 | monoacylglycerol O-acyltransferase 2 |
| 96 | MIMAT0001884,MIMAT0001816 | 0.04744 | 555814 | zgc:174354 | zgc:174354 |
| 97 | MIMAT0001836,MIMAT0001852 | 0.04744 | 563215 | vps41 | vacuolar protein sorting 41 homolog (S. cerevisiae) |
| 98 | MIMAT0001852,MIMAT0001789 | 0.04744 | 594858 | hamp2 | hepcidin antimicrobial peptide 2 |
| 99 | MIMAT0001870,MIMAT0001836,MIMAT0001816 | 0.04782 | 100003562 | ecd | ecdysoneless homolog (Drosophila) |
| 100 | MIMAT0001836,MIMAT0001852,MIMAT0001816 | 0.04782 | 100148840 | wnt7bb | wingless-type MMTV integration site family, member 7Bb |
| 101 | MIMAT0001884,MIMAT0001870,MIMAT0001852 | 0.04782 | 192305 | adssl | adenylosuccinate synthase, like |
| 102 | MIMAT0001884,MIMAT0001789,MIMAT0001852 | 0.04782 | 30038 | sox19a | SRY-box containing gene 19a |
| 103 | MIMAT0001884,MIMAT0001836,MIMAT0001852 | 0.04782 | 503747 | fitm1 | fat storage-inducing transmembrane protein 1 |
| 104 | MIMAT0001762 | 0.04838 | 100001198 | prkaa1 | protein kinase, AMP-activated, alpha 1 catalytic subunit |
| 105 | MIMAT0001870,MIMAT0001852,MIMAT0001816,MIMAT0001789 | 0.04951 | 100141360 | btbd6a | BTB (POZ) domain containing 6a |
| 106 | MIMAT0001870,MIMAT0001836,MIMAT0001852,MIMAT0001884 | 0.04951 | 337436 | dennd6b | DENN/MADD domain containing 6B |
| 107 | MIMAT0001870,MIMAT0001852,MIMAT0001816,MIMAT0001884 | 0.04951 | 406602 | egln3 | egl nine homolog 3 (C. elegans) |
| 108 | MIMAT0001870,MIMAT0001852,MIMAT0001816,MIMAT0001884 | 0.04951 | 541506 | zgc:113227 | zgc:113227 |
| 109 | MIMAT0001836,MIMAT0001856,MIMAT0001884,MIMAT0001762 | 0.04951 | 565192 | gpr146 | G protein-coupled receptor 146 |

Table S2: Significantly (>2.0 fold) up-regulated miRNA in the liver of zebrafish fasted and exposed to fluoxetine (540 ng/L) for 7 days and their *in silico* predicted target genes. Target genes are sorted by ascending level of significant likelihood of targeting said gene. Legend: MIMAT0001762 = dre-let-7d; MIMAT0001789 = dre-miR-22b; MIMAT0001836 = dre-miR-140-5p; MIMAT0001856 = dre-miR-193a; MIMAT0001870 = dre-miR-301a; MIMAT0001884 = dre-miR-457b.

|  | **mirbase accession No** | **p-value** | **GeneID** | **Symbol** | **Description** |
| --- | --- | --- | --- | --- | --- |
| 1 | MIMAT0001870,MIMAT0001836,MIMAT0001856,MIMAT0001884,MIMAT0001762 | 0.00122 | 497350 | im:7137555 | im:7137555 |
| 2 | MIMAT0001870,MIMAT0001836,MIMAT0001856,MIMAT0001884,MIMAT0001789 | 0.00239 | 541316 | lrrc1 | leucine rich repeat containing 1 |
| 3 | MIMAT0001836,MIMAT0001856,MIMAT0001884,MIMAT0001789 | 0.00892 | 100005490 | zrsr2 | zinc finger (CCCH type), RNA-binding motif and serine/arginine rich 2 |
| 4 | MIMAT0001870,MIMAT0001856,MIMAT0001884,MIMAT0001789 | 0.00892 | 562525 | cldn2 | claudin 2 |
| 5 | MIMAT0001856,MIMAT0001789 | 0.01297 | 562337 | si:dkeyp-53d3.3 | si:dkeyp-53d3.3 |
| 6 | MIMAT0001836,MIMAT0001856,MIMAT0001884,MIMAT0001762 | 0.01446 | 565192 | gpr146 | G protein-coupled receptor 146 |
| 7 | MIMAT0001870,MIMAT0001836,MIMAT0001884,MIMAT0001789,MIMAT0001762 | 0.01841 | 406717 | slc25a22 | solute carrier family 25 (mitochondrial carrier: glutamate), member 22 |
| 8 | MIMAT0001836,MIMAT0001856 | 0.02167 | 405880 | zgc:85843 | zgc:85843 |
| 9 | MIMAT0001870,MIMAT0001836,MIMAT0001789,MIMAT0001856,MIMAT0001762 | 0.02227 | 565854 | onecut3 | one cut homeobox 3 |
| 10 | MIMAT0001870,MIMAT0001836,MIMAT0001789 | 0.02501 | 30537 | emx2 | empty spiracles homeobox 2 |
| 11 | MIMAT0001870,MIMAT0001856,MIMAT0001884 | 0.02501 | 406271 | etfb | electron-transfer-flavoprotein, beta polypeptide |
| 12 | MIMAT0001836,MIMAT0001856,MIMAT0001884 | 0.02501 | 562912 | ltbp3 | latent transforming growth factor beta binding protein 3 |
| 13 | MIMAT0001836 | 0.02804 | 572074 | prkaa2 | protein kinase, AMP-activated, alpha 2 catalytic subunit |
| 14 | MIMAT0001836 | 0.02804 | 678590 | zgc:136254 | zgc:136254 |
| 15 | MIMAT0001870,MIMAT0001884,MIMAT0001789,MIMAT0001856 | 0.02977 | 558048 | n4bp2 | NEDD4 binding protein 2 |
| 16 | MIMAT0001836,MIMAT0001789 | 0.03227 | 322462 | sult6b1 | sulfotransferase family, cytosolic, 6b, member 1 |
| 17 | MIMAT0001836,MIMAT0001884,MIMAT0001789 | 0.03395 | 556582 | grik4 | glutamate receptor, ionotropic, kainate 4 |
| 18 | MIMAT0001870,MIMAT0001836,MIMAT0001884 | 0.03395 | 79377 | gfra1b | gdnf family receptor alpha 1b |
| 19 | MIMAT0001836,MIMAT0001884,MIMAT0001789 | 0.03729 | 561938 | thumpd3 | THUMP domain containing 3 |
| 20 | MIMAT0001836,MIMAT0001856 | 0.03823 | 30658 | rag2 | recombination activating gene 2 |
| 21 | MIMAT0001884,MIMAT0001836 | 0.03823 | 562262 | tnfsf10l3 | tumor necrosis factor (ligand) superfamily, member 10 like 3 |
| 22 | MIMAT0001836,MIMAT0001789 | 0.03823 | 768137 | dclre1b | DNA cross-link repair 1B |
| 23 | MIMAT0001836,MIMAT0001856,MIMAT0001762 | 0.04081 | 100301946 | igfbp6b | insulin-like growth factor binding protein 6b |
| 24 | MIMAT0001870,MIMAT0001856,MIMAT0001789 | 0.04081 | 677750 | myl2b | myosin, light polypeptide 2b, regulatory, cardiac, slow |
| 25 | MIMAT0001870,MIMAT0001884,MIMAT0001789,MIMAT0001856 | 0.04177 | 30181 | ptch2 | patched 2 |
| 26 | MIMAT0001870,MIMAT0001836,MIMAT0001884,MIMAT0001856 | 0.04177 | 557456 | trpc5a | transient receptor potential cation channel, subfamily C, member 5a |
| 27 | MIMAT0001870,MIMAT0001884,MIMAT0001856,MIMAT0001762 | 0.04450 | 64812 | sox19b | SRY-box containing gene 19b |
| 28 | MIMAT0001870,MIMAT0001836,MIMAT0001789 | 0.04450 | 555510 | cyp2aa12 | cytochrome P450, family 2, subfamily AA, polypeptide 12 |
| 29 | MIMAT0001884,MIMAT0001856 | 0.04460 | 327449 | rasa2 | RAS p21 protein activator 2 |
| 30 | MIMAT0001836,MIMAT0001884,MIMAT0001789 | 0.04838 | 100320541 | spock2 | sparc/osteonectin, cwcv and kazal-like domains proteoglycan (testican) 2 |
| 31 | MIMAT0001762 | 0.04838 | 100001198 | prkaa1 | protein kinase, AMP-activated, alpha 1 catalytic subunit |

Table S3: Significantly (>2.0 fold) up-regulated miRNA in the liver of zebrafish fed and exposed to fluoxetine (540 ng/L) for 7 days and their *in silico* predicted target genes. Target genes are sorted by ascending level of significant likelihood of targeting said gene. Legend: MIMAT0001762 = dre-let-7d; MIMAT0001767 = dre-let-7i; MIMAT0001789 = dre-miR-22b; MIMAT0001836 = dre-miR-140-5p; MIMAT0001852 = dre-miR-181c; MIMAT0001856 = dre-miR-193a; MIMAT0001870 = dre-miR-301a; MIMAT0001884 = dre-miR-457b.

|  | **mirbase accession No** | **p-value** | **GeneID** | **Symbol** | **Description** |
| --- | --- | --- | --- | --- | --- |
| 1 | MIMAT0001870,MIMAT0001836,MIMAT0001852,MIMAT0001884,MIMAT0001762,MIMAT0001767 | 0.00023 | 497350 | im:7137555 | im:7137555 |
| 2 | MIMAT0001836,MIMAT0001852,MIMAT0001789 | 0.00319 | 322462 | sult6b1 | sulfotransferase family, cytosolic, 6b, member 1 |
| 3 | MIMAT0001870,MIMAT0001884,MIMAT0001789,MIMAT0001852,MIMAT0001762,MIMAT0001767 | 0.00321 | 368893 | acin1a | apoptotic chromatin condensation inducer 1a |
| 4 | MIMAT0001836,MIMAT0001884,MIMAT0001789,MIMAT0001852,MIMAT0001762,MIMAT0001767 | 0.00459 | 436796 | rgs17 | regulator of G-protein signaling 17 |
| 5 | MIMAT0001870,MIMAT0001884,MIMAT0001789,MIMAT0001852,MIMAT0001762,MIMAT0001767 | 0.00459 | 569067 | faah | fatty acid amide hydrolase |
| 6 | MIMAT0001870,MIMAT0001836,MIMAT0001852,MIMAT0001789 | 0.00474 | 30537 | emx2 | empty spiracles homeobox 2 |
| 7 | MIMAT0001852,MIMAT0001762,MIMAT0001789,MIMAT0001767 | 0.00549 | 406288 | srsf1a | serine/arginine-rich splicing factor 1a |
| 8 | MIMAT0001870,MIMAT0001836,MIMAT0001852,MIMAT0001762,MIMAT0001767 | 0.00565 | 563087 | si:ch211-129c21.1 | si:ch211-129c21.1 |
| 9 | MIMAT0001852,MIMAT0001884,MIMAT0001762,MIMAT0001789,MIMAT0001767 | 0.00565 | 569471 | chd7 | chromodomain helicase DNA binding protein 7 |
| 10 | MIMAT0001870,MIMAT0001836,MIMAT0001884,MIMAT0001789,MIMAT0001762,MIMAT0001767 | 0.00694 | 406717 | slc25a22 | solute carrier family 25 (mitochondrial carrier: glutamate), member 22 |
| 11 | MIMAT0001836,MIMAT0001852,MIMAT0001762,MIMAT0001767 | 0.00826 | 559078 | si:dkey-238c7.16 | si:dkey-238c7.16 |
| 12 | MIMAT0001762,MIMAT0001767,MIMAT0001836 | 0.00830 | 393843 | zgc:77816 | zgc:77816 |
| 13 | MIMAT0001762,MIMAT0001767,MIMAT0001852 | 0.00830 | 406267 | hnrnpk | heterogeneous nuclear ribonucleoprotein K |
| 14 | MIMAT0001870,MIMAT0001836,MIMAT0001789,MIMAT0001852,MIMAT0001762,MIMAT0001767 | 0.00876 | 565854 | onecut3 | one cut homeobox 3 |
| 15 | MIMAT0001870,MIMAT0001852,MIMAT0001762,MIMAT0001767 | 0.00936 | 393882 | tubg1 | tubulin, gamma 1 |
| 16 | MIMAT0001884,MIMAT0001789,MIMAT0001852,MIMAT0001762,MIMAT0001767 | 0.01011 | 334003 | pho | phoenix |
| 17 | MIMAT0001852,MIMAT0001762,MIMAT0001789,MIMAT0001767 | 0.01057 | 30467 | max | myc-associated factor X |
| 18 | MIMAT0001852,MIMAT0001762,MIMAT0001789,MIMAT0001767 | 0.01057 | 554107 | ela3l | elastase 3 like |
| 19 | MIMAT0001762,MIMAT0001789,MIMAT0001767 | 0.01205 | 568941 | oc90 | otoconin 90 |
| 20 | MIMAT0001884,MIMAT0001789,MIMAT0001852,MIMAT0001762,MIMAT0001767 | 0.01206 | 100002125 | tacr2 | tachykinin receptor 2 |
| 21 | MIMAT0001870,MIMAT0001884,MIMAT0001852,MIMAT0001762,MIMAT0001767 | 0.01206 | 407654 | mob1bb | MOB kinase activator 1Bb |
| 22 | MIMAT0001836,MIMAT0001884,MIMAT0001852,MIMAT0001762,MIMAT0001767 | 0.01206 | 431773 | enoph1 | enolase-phosphatase 1 |
| 23 | MIMAT0001836,MIMAT0001789,MIMAT0001852,MIMAT0001762,MIMAT0001767 | 0.01313 | 30771 | hic1l | hypermethylated in cancer 1 like |
| 24 | MIMAT0001852,MIMAT0001884,MIMAT0001762,MIMAT0001767 | 0.01328 | 692316 | atxn7l2a | ataxin 7-like 2a |
| 25 | MIMAT0001836,MIMAT0001884,MIMAT0001852,MIMAT0001762,MIMAT0001767 | 0.01428 | 548341 | nr1h3 | nuclear receptor subfamily 1, group H, member 3 |
| 26 | MIMAT0001884,MIMAT0001789,MIMAT0001852,MIMAT0001762,MIMAT0001767 | 0.01428 | 559332 | pde6b | phosphodiesterase 6B, cGMP-specific, rod, beta |
| 27 | MIMAT0001870,MIMAT0001884,MIMAT0001852,MIMAT0001762,MIMAT0001767 | 0.01550 | 405878 | fbxl15 | F-box and leucine-rich repeat protein 15 |
| 28 | MIMAT0001870,MIMAT0001836,MIMAT0001884,MIMAT0001852,MIMAT0001762,MIMAT0001767 | 0.01553 | 260350 | has2 | hyaluronan synthase 2 |
| 29 | MIMAT0001836,MIMAT0001884,MIMAT0001762,MIMAT0001767 | 0.01644 | 436928 | atxn7l2b | ataxin 7-like 2b |
| 30 | MIMAT0001870,MIMAT0001852,MIMAT0001762,MIMAT0001767 | 0.01644 | 555286 | tox3 | TOX high mobility group box family member 3 |
| 31 | MIMAT0001870,MIMAT0001884,MIMAT0001789,MIMAT0001852,MIMAT0001762,MIMAT0001767 | 0.01661 | 550555 | slc35e4 | solute carrier family 35, member E4 |
| 32 | MIMAT0001789,MIMAT0001836,MIMAT0001852 | 0.01666 | 405810 | ehd1a | EH-domain containing 1a |
| 33 | MIMAT0001870,MIMAT0001836,MIMAT0001884,MIMAT0001762,MIMAT0001767 | 0.01816 | 553355 | col4a2 | collagen, type IV, alpha 2 |
| 34 | MIMAT0001870,MIMAT0001884,MIMAT0001762,MIMAT0001767 | 0.01820 | 405787 | fibpb | fibroblast growth factor (acidic) intracellular binding protein b |
| 35 | MIMAT0001870,MIMAT0001836,MIMAT0001762,MIMAT0001767 | 0.01820 | 565976 | zgc:114118 | zgc:114118 |
| 36 | MIMAT0001870,MIMAT0001852,MIMAT0001762,MIMAT0001767 | 0.01820 | 79079 | zgc:194449 | zgc:194449 |
| 37 | MIMAT0001762,MIMAT0001767,MIMAT0001852 | 0.01931 | 322020 | wu:fb44b02 | wu:fb44b02 |
| 38 | MIMAT0001884,MIMAT0001789,MIMAT0001852,MIMAT0001762,MIMAT0001767 | 0.01961 | 245698 | flot2a | flotillin 2a |
| 39 | MIMAT0001884,MIMAT0001789,MIMAT0001852,MIMAT0001762,MIMAT0001767 | 0.01961 | 550262 | h3f3b.1 | H3 histone, family 3B.1 |
| 40 | MIMAT0001870,MIMAT0001789,MIMAT0001852,MIMAT0001762,MIMAT0001767 | 0.01961 | 560100 | adora2b | adenosine A2b receptor |
| 41 | MIMAT0001852,MIMAT0001762,MIMAT0001789,MIMAT0001767 | 0.02008 | 553360 | pcif1 | PDX1 C-terminal inhibiting factor 1 |
| 42 | MIMAT0001836,MIMAT0001884,MIMAT0001852,MIMAT0001762,MIMAT0001767 | 0.02115 | 564996 | tfe3b | transcription factor binding to IGHM enhancer 3b |
| 43 | MIMAT0001836,MIMAT0001884,MIMAT0001852,MIMAT0001762,MIMAT0001767 | 0.02115 | 569093 | zgc:114104 | zgc:114104 |
| 44 | MIMAT0001836,MIMAT0001884,MIMAT0001852,MIMAT0001762,MIMAT0001767 | 0.02115 | 768157 | cplx4a | complexin 4a |
| 45 | MIMAT0001852,MIMAT0001762,MIMAT0001789,MIMAT0001767 | 0.02209 | 323226 | wsb1 | WD repeat and SOCS box-containing 1 |
| 46 | MIMAT0001836,MIMAT0001884,MIMAT0001762,MIMAT0001767 | 0.02209 | 334618 | eif4g2b | eukaryotic translation initiation factor 4, gamma 2b |
| 47 | MIMAT0001836,MIMAT0001852,MIMAT0001762,MIMAT0001767 | 0.02209 | 406713 | lipf | lipase, gastric |
| 48 | MIMAT0001836,MIMAT0001852,MIMAT0001762,MIMAT0001767 | 0.02209 | 58090 | trpc4apa | transient receptor potential cation channel, subfamily C, member 4 associated protein a |
| 49 | MIMAT0001789,MIMAT0001836,MIMAT0001852 | 0.02219 | 325469 | fbxo9 | F-box protein 9 |
| 50 | MIMAT0001762,MIMAT0001767,MIMAT0001852 | 0.02219 | 550353 | histh1l | histone H1 like |
| 51 | MIMAT0001762,MIMAT0001767,MIMAT0001836 | 0.02219 | 561841 | wu:fb39e10 | wu:fb39e10 |
| 52 | MIMAT0001762,MIMAT0001767,MIMAT0001852 | 0.02219 | 564554 | lrig3 | leucine-rich repeats and immunoglobulin-like domains 3 |
| 53 | MIMAT0001870,MIMAT0001836,MIMAT0001884,MIMAT0001762,MIMAT0001767 | 0.02277 | 436745 | atp2b3a | ATPase, Ca++ transporting, plasma membrane 3a |
| 54 | MIMAT0001884,MIMAT0001789,MIMAT0001852,MIMAT0001762,MIMAT0001767 | 0.02277 | 558652 | sp3a | sp3a transcription factor |
| 55 | MIMAT0001836,MIMAT0001884,MIMAT0001852,MIMAT0001762,MIMAT0001767 | 0.02277 | 678518 | baiap2a | BAI1-associated protein 2a |
| 56 | MIMAT0001870,MIMAT0001836,MIMAT0001852,MIMAT0001762,MIMAT0001767 | 0.02448 | 393953 | prkcbb | protein kinase C, beta b |
| 57 | MIMAT0001762,MIMAT0001789,MIMAT0001767 | 0.02531 | 100007842 | zgc:174314 | zgc:174314 |
| 58 | MIMAT0001762,MIMAT0001767,MIMAT0001836 | 0.02531 | 555180 | zp2l1 | zona pellucida glycoprotein 2, like 1 |
| 59 | MIMAT0001762,MIMAT0001767,MIMAT0001836 | 0.02531 | 792319 | saga | S-antigen; retina and pineal gland (arrestin) a |
| 60 | MIMAT0001870,MIMAT0001836,MIMAT0001852,MIMAT0001762,MIMAT0001767 | 0.02628 | 393300 | gbgt1l1 | globoside alpha-1,3-N-acetylgalactosaminyltransferase 1, like 1 |
| 61 | MIMAT0001836,MIMAT0001852,MIMAT0001762,MIMAT0001767 | 0.02649 | 192309 | tpi1a | triosephosphate isomerase 1a |
| 62 | MIMAT0001836,MIMAT0001762,MIMAT0001789,MIMAT0001767 | 0.02649 | 368213 | glo1 | glyoxalase 1 |
| 63 | MIMAT0001836,MIMAT0001852,MIMAT0001762,MIMAT0001767 | 0.02649 | 405841 | zgc:77086 | zgc:77086 |
| 64 | MIMAT0001852,MIMAT0001884,MIMAT0001762,MIMAT0001767 | 0.02649 | 565672 | rnpc3 | RNA-binding region (RNP1, RRM) containing 3 |
| 65 | MIMAT0001836,MIMAT0001852,MIMAT0001762,MIMAT0001767 | 0.02649 | 792610 | hspb6 | heat shock protein, alpha-crystallin-related, b6 |
| 66 | MIMAT0001836 | 0.02804 | 572074 | prkaa2 | protein kinase, AMP-activated, alpha 2 catalytic subunit |
| 67 | MIMAT0001836,MIMAT0001789,MIMAT0001852,MIMAT0001762,MIMAT0001767 | 0.02818 | 100003342 | il12rb2l | interleukin 12 receptor, beta 2a, like |
| 68 | MIMAT0001884,MIMAT0001789,MIMAT0001852,MIMAT0001762,MIMAT0001767 | 0.02818 | 30222 | ccnd1 | cyclin D1 |
| 69 | MIMAT0001762,MIMAT0001789,MIMAT0001767 | 0.02867 | 100007972 | zgc:173705 | zgc:173705 |
| 70 | MIMAT0001762,MIMAT0001767,MIMAT0001836 | 0.02867 | 30405 | hoxd11a | homeo box D11a |
| 71 | MIMAT0001884,MIMAT0001870,MIMAT0001852 | 0.02867 | 550376 | cib1 | calcium and integrin binding 1 (calmyrin) |
| 72 | MIMAT0001870,MIMAT0001789,MIMAT0001852 | 0.02867 | 558261 | ociad2 | OCIA domain containing 2 |
| 73 | MIMAT0001870,MIMAT0001836,MIMAT0001852,MIMAT0001884 | 0.02889 | 337436 | dennd6b | DENN/MADD domain containing 6B |
| 74 | MIMAT0001836,MIMAT0001884,MIMAT0001762,MIMAT0001767 | 0.02889 | 565192 | gpr146 | G protein-coupled receptor 146 |
| 75 | MIMAT0001870,MIMAT0001884,MIMAT0001852,MIMAT0001762,MIMAT0001767 | 0.03017 | 572463 | ibtk | inhibitor of Bruton agammaglobulinemia tyrosine kinase |
| 76 | MIMAT0001852,MIMAT0001884,MIMAT0001762,MIMAT0001767 | 0.03144 | 568429 | trove2 | TROVE domain family, member 2 |
| 77 | MIMAT0001870,MIMAT0001884,MIMAT0001852,MIMAT0001762,MIMAT0001767 | 0.03227 | 100333407 | adamts9 | ADAM metallopeptidase with thrombospondin type 1 motif, 9 |
| 78 | MIMAT0001870,MIMAT0001836,MIMAT0001852,MIMAT0001762,MIMAT0001767 | 0.03227 | 60641 | sox9a | SRY-box containing gene 9a |
| 79 | MIMAT0001884,MIMAT0001870,MIMAT0001852 | 0.03228 | 192305 | adssl | adenylosuccinate synthase, like |
| 80 | MIMAT0001884,MIMAT0001789,MIMAT0001852 | 0.03228 | 30038 | sox19a | SRY-box containing gene 19a |
| 81 | MIMAT0001762,MIMAT0001767,MIMAT0001852 | 0.03228 | 368823 | fkbp11 | FK506 binding protein 11 |
| 82 | MIMAT0001884,MIMAT0001836,MIMAT0001852 | 0.03228 | 503747 | fitm1 | fat storage-inducing transmembrane protein 1 |
| 83 | MIMAT0001789,MIMAT0001836,MIMAT0001852 | 0.03228 | 555279 | tmem209 | transmembrane protein 209 |
| 84 | MIMAT0001762,MIMAT0001767,MIMAT0001852 | 0.03228 | 562261 | aig1 | androgen-induced 1 (H. sapiens) |
| 85 | MIMAT0001836 | 0.03271 | 678590 | zgc:136254 | zgc:136254 |
| 86 | MIMAT0001870,MIMAT0001836,MIMAT0001762,MIMAT0001767 | 0.03413 | 393970 | acy1 | aminoacylase 1 |
| 87 | MIMAT0001852,MIMAT0001884,MIMAT0001762,MIMAT0001767 | 0.03413 | 406512 | nfe2l3 | nuclear factor (erythroid-derived 2)-like 3 |
| 88 | MIMAT0001870,MIMAT0001852,MIMAT0001762,MIMAT0001767 | 0.03413 | 799880 | nwd1 | NACHT and WD repeat domain containing 1 |
| 89 | MIMAT0001870,MIMAT0001884,MIMAT0001852,MIMAT0001762,MIMAT0001767 | 0.03447 | 322903 | wu:fb76c12 | wu:fb76c12 |
| 90 | MIMAT0001870,MIMAT0001884,MIMAT0001852,MIMAT0001762,MIMAT0001767 | 0.03447 | 554960 | ubxn6 | UBX domain protein 6 |
| 91 | MIMAT0001852,MIMAT0001884,MIMAT0001789 | 0.03613 | 571289 | gsdf | gonadal somatic cell derived factor |
| 92 | MIMAT0001852,MIMAT0001789 | 0.03650 | 406266 | rsl24d1 | ribosomal L24 domain containing 1 |
| 93 | MIMAT0001767,MIMAT0001762 | 0.03650 | 415098 | top1mt | mitochondrial topoisomerase I |
| 94 | MIMAT0001767,MIMAT0001762 | 0.03650 | 445028 | zgc:92237 | zgc:92237 |
| 95 | MIMAT0001836,MIMAT0001852 | 0.03650 | 450063 | mogat2 | monoacylglycerol O-acyltransferase 2 |
| 96 | MIMAT0001836,MIMAT0001852 | 0.03650 | 563215 | vps41 | vacuolar protein sorting 41 homolog (S. cerevisiae) |
| 97 | MIMAT0001852,MIMAT0001789 | 0.03650 | 594858 | hamp2 | hepcidin antimicrobial peptide 2 |
| 98 | MIMAT0001870,MIMAT0001884,MIMAT0001852,MIMAT0001762,MIMAT0001767 | 0.03677 | 321807 | eif2s1a | eukaryotic translation initiation factor 2, subunit 1 alpha a |
| 99 | MIMAT0001870,MIMAT0001852,MIMAT0001762,MIMAT0001767 | 0.03696 | 246225 | onecutl | one cut domain, family member, like |
| 100 | MIMAT0001852,MIMAT0001884,MIMAT0001762,MIMAT0001767 | 0.03696 | 378875 | tsc22d2 | TSC22 domain family 2 |
| 101 | MIMAT0001852,MIMAT0001884,MIMAT0001762,MIMAT0001767 | 0.03696 | 386644 | ddi2 | DNA-damage inducible protein 2 |
| 102 | MIMAT0001870,MIMAT0001852,MIMAT0001762,MIMAT0001767 | 0.03696 | 393634 | trappc6bl | trafficking protein particle complex 6b-like |
| 103 | MIMAT0001870,MIMAT0001852,MIMAT0001762,MIMAT0001767 | 0.03696 | 560592 | myo15aa | myosin XVAa |
| 104 | MIMAT0001870,MIMAT0001884,MIMAT0001762,MIMAT0001767 | 0.03696 | 796380 | eif3ba | eukaryotic translation initiation factor 3, subunit Ba |
| 105 | MIMAT0001836,MIMAT0001884,MIMAT0001852,MIMAT0001762,MIMAT0001767 | 0.03918 | 100141327 | igdcc4 | immunoglobulin superfamily, DCC subclass, member 4 |
| 106 | MIMAT0001836,MIMAT0001852,MIMAT0001762,MIMAT0001767 | 0.03994 | 30473 | rx2 | retinal homeobox gene 2 |
| 107 | MIMAT0001870,MIMAT0001836,MIMAT0001852,MIMAT0001789 | 0.03994 | 334490 | hnrnpub | heterogeneous nuclear ribonucleoprotein Ub |
| 108 | MIMAT0001870,MIMAT0001852,MIMAT0001762,MIMAT0001767 | 0.03994 | 334749 | seh1l | SEH1-like (S. cerevisiae) |
| 109 | MIMAT0001870,MIMAT0001762,MIMAT0001789,MIMAT0001767 | 0.03994 | 406756 | tmem129 | transmembrane protein 129 |
| 110 | MIMAT0001870,MIMAT0001836,MIMAT0001762,MIMAT0001767 | 0.03994 | 555974 | mknk1 | MAP kinase interacting serine/threonine kinase 1 |
| 111 | MIMAT0001870,MIMAT0001836,MIMAT0001762,MIMAT0001767 | 0.03994 | 566608 | upf3a | UPF3 regulator of nonsense transcripts homolog A (yeast) |
| 112 | MIMAT0001870,MIMAT0001852,MIMAT0001762,MIMAT0001767 | 0.03994 | 58057 | pou4f1 | POU domain, class 4, transcription factor 1 |
| 113 | MIMAT0001762,MIMAT0001789,MIMAT0001767 | 0.04022 | 100126112 | zgc:173714 | zgc:173714 |
| 114 | MIMAT0001762,MIMAT0001789,MIMAT0001767 | 0.04022 | 327206 | immt | inner membrane protein, mitochondrial (mitofilin) |
| 115 | MIMAT0001870,MIMAT0001836,MIMAT0001852 | 0.04022 | 569420 | wnt3 | wingless-type MMTV integration site family, member 3 |
| 116 | MIMAT0001836,MIMAT0001762,MIMAT0001767 | 0.04022 | 613239 | hcrt | hypocretin (orexin) neuropeptide precursor |
| 117 | MIMAT0001870,MIMAT0001884,MIMAT0001789,MIMAT0001762,MIMAT0001767 | 0.04170 | 324384 | syncripl | synaptotagmin binding, cytoplasmic RNA interacting protein, like |
| 118 | MIMAT0001852,MIMAT0001884,MIMAT0001762,MIMAT0001767 | 0.04308 | 100526672 | ptprsb | protein tyrosine phosphatase, receptor type, s, b |
| 119 | MIMAT0001852,MIMAT0001884,MIMAT0001762,MIMAT0001767 | 0.04308 | 30560 | fgf6a | fibroblast growth factor 6a |
| 120 | MIMAT0001870,MIMAT0001852,MIMAT0001762,MIMAT0001767 | 0.04308 | 30702 | zorba | Orb/CPEB-related RNA-binding protein |
| 121 | MIMAT0001836,MIMAT0001852,MIMAT0001762,MIMAT0001767 | 0.04308 | 559491 | tmem160 | transmembrane protein 160 |
| 122 | MIMAT0001870,MIMAT0001836,MIMAT0001852,MIMAT0001884 | 0.04308 | 569727 | phip | pleckstrin homology domain interacting protein |
| 123 | MIMAT0001836,MIMAT0001852,MIMAT0001762,MIMAT0001767 | 0.04308 | 571915 | si:dkey-9i23.16 | si:dkey-9i23.16 |
| 124 | MIMAT0001836,MIMAT0001852 | 0.04390 | 100141499 | zgc:174698 | zgc:174698 |
| 125 | MIMAT0001767,MIMAT0001762 | 0.04390 | 403061 | ela2 | elastase 2 |
| 126 | MIMAT0001852,MIMAT0001789 | 0.04390 | 415158 | cycsb | cytochrome c, somatic b |
| 127 | MIMAT0001852,MIMAT0001789 | 0.04390 | 445046 | hars2 | histidyl-tRNA synthetase 2, mitochondrial (putative) |
| 128 | MIMAT0001767,MIMAT0001762 | 0.04390 | 556456 | pold1 | polymerase (DNA directed), delta 1, catalytic subunit |
| 129 | MIMAT0001836,MIMAT0001884,MIMAT0001852,MIMAT0001762,MIMAT0001767 | 0.04433 | 553337 | lamb2l | laminin, beta 2-like |
| 130 | MIMAT0001836,MIMAT0001789,MIMAT0001852,MIMAT0001762,MIMAT0001767 | 0.04433 | 558181 | ctdspl2a | CTD (carboxy-terminal domain, RNA polymerase II, polypeptide A) small phosphatase like 2a |
| 131 | MIMAT0001836,MIMAT0001762,MIMAT0001767 | 0.04456 | 317764 | azin1b | antizyme inhibitor 1b |
| 132 | MIMAT0001852,MIMAT0001884,MIMAT0001789 | 0.04456 | 324510 | gcnt4a | glucosaminyl (N-acetyl) transferase 4, core 2, a |
| 133 | MIMAT0001884,MIMAT0001762,MIMAT0001767 | 0.04456 | 334717 | banf1 | barrier to autointegration factor 1 |
| 134 | MIMAT0001852,MIMAT0001762,MIMAT0001767 | 0.04456 | 403082 | sh3bp4a | SH3-domain binding protein 4a |
| 135 | MIMAT0001852,MIMAT0001762,MIMAT0001767 | 0.04456 | 445100 | ube2e2 | ubiquitin-conjugating enzyme E2E 2 |
| 136 | MIMAT0001884,MIMAT0001762,MIMAT0001767 | 0.04456 | 553420 | mipb | major intrinsic protein of lens fiber b |
| 137 | MIMAT0001836,MIMAT0001762,MIMAT0001767 | 0.04456 | 751625 | mettl21d | methyltransferase like 21D |
| 138 | MIMAT0001870,MIMAT0001884,MIMAT0001762,MIMAT0001767 | 0.04636 | 30138 | dld | deltaD |
| 139 | MIMAT0001870,MIMAT0001852,MIMAT0001884,MIMAT0001789 | 0.04636 | 393187 | atp5s | ATP synthase, H+ transporting, mitochondrial F0 complex, subunit s |
| 140 | MIMAT0001870,MIMAT0001852,MIMAT0001762,MIMAT0001767 | 0.04636 | 407659 | st3gal3b | ST3 beta-galactoside alpha-2,3-sialyltransferase 3b |
| 141 | MIMAT0001836,MIMAT0001852,MIMAT0001762,MIMAT0001767 | 0.04636 | 492354 | fam83fb | family with sequence similarity 83, member Fb |
| 142 | MIMAT0001836,MIMAT0001762,MIMAT0001789,MIMAT0001767 | 0.04636 | 492363 | slc7a3a | solute carrier family 7 (cationic amino acid transporter, y+ system), member 3a |
| 143 | MIMAT0001870,MIMAT0001836,MIMAT0001884,MIMAT0001789 | 0.04636 | 541316 | lrrc1 | leucine rich repeat containing 1 |
| 144 | MIMAT0001852,MIMAT0001884,MIMAT0001762,MIMAT0001767 | 0.04636 | 541488 | tcp11l1 | t-complex 11 (mouse)-like 1 |
| 145 | MIMAT0001852,MIMAT0001762,MIMAT0001789,MIMAT0001767 | 0.04636 | 566825 | mll4b | myeloid/lymphoid or mixed-lineage leukemia 4b |
| 146 | MIMAT0001870,MIMAT0001836,MIMAT0001852,MIMAT0001789 | 0.04636 | 568410 | zgc:194990 | zgc:194990 |
| 147 | MIMAT0001870,MIMAT0001836,MIMAT0001852,MIMAT0001884 | 0.04636 | 572204 | ngfr | nerve growth factor receptor (TNFR superfamily, member 16) |
| 148 | MIMAT0001870,MIMAT0001884,MIMAT0001852,MIMAT0001762,MIMAT0001767 | 0.04708 | 324335 | usp19 | ubiquitin specific peptidase 19 |
| 149 | MIMAT0001870,MIMAT0001884,MIMAT0001852,MIMAT0001762,MIMAT0001767 | 0.04708 | 562720 | mapk8a | mitogen-activated protein kinase 8a |
| 150 | MIMAT0001762 | 0.04838 | 100001198 | prkaa1 | protein kinase, AMP-activated, alpha 1 catalytic subunit |
| 151 | MIMAT0001870,MIMAT0001762,MIMAT0001767 | 0.04915 | 252846 | myo7aa | myosin VIIAa |
| 152 | MIMAT0001852,MIMAT0001762,MIMAT0001767 | 0.04915 | 492344 | cyp1d1 | cytochrome P450, family 1, subfamily D, polypeptide 1 |
| 153 | MIMAT0001870,MIMAT0001852,MIMAT0001884 | 0.04915 | 555285 | myo1e | myosin IE |
| 154 | MIMAT0001870,MIMAT0001762,MIMAT0001767 | 0.04915 | 563729 | samd7 | sterile alpha motif domain containing 7 |
| 155 | MIMAT0001836,MIMAT0001852,MIMAT0001762,MIMAT0001767 | 0.04981 | 327294 | gatsl2 | GATS protein-like 2 |
| 156 | MIMAT0001836,MIMAT0001852,MIMAT0001762,MIMAT0001767 | 0.04981 | 436673 | gng13b | guanine nucleotide binding protein (G protein), gamma 13b |
| 157 | MIMAT0001836,MIMAT0001852,MIMAT0001762,MIMAT0001767 | 0.04981 | 550515 | crlf3 | cytokine receptor-like factor 3 |
| 158 | MIMAT0001836,MIMAT0001884,MIMAT0001789,MIMAT0001852 | 0.04981 | 553226 | tmem198a | transmembrane protein 198a |
| 159 | MIMAT0001836,MIMAT0001884,MIMAT0001789,MIMAT0001762,MIMAT0001767 | 0.04995 | 324832 | sec22a | SEC22 vesicle trafficking protein homolog A (S. cerevisiae) |
| 160 | MIMAT0001884,MIMAT0001789,MIMAT0001852,MIMAT0001762,MIMAT0001767 | 0.04995 | 567154 | sox6 | SRY-box containing gene 6 |

Table S4: Significantly (>2.0 fold) down-regulated miRNA in the liver of zebrafish fasted and exposed to fluoxetine (540 ng/L) for 7 days and their *in silico* predicted target genes. Target genes are sorted by ascending level of significant likelihood of targeting said gene. Legend: MIMAT0001280 = dre-miR-205.

|  | **mirbase accession No** | **p-value** | **GeneID** | **Symbol** | **Description** |
| --- | --- | --- | --- | --- | --- |
| 1 | MIMAT0001280 | 0.04673 | 100003014 | zgc:153292 | zgc:153292 |
| 2 | MIMAT0001280 | 0.04673 | 100003691 | socs9 | suppressor of cytokine signaling 9 |
| 3 | MIMAT0001280 | 0.04206 | 100006734 | si:dkey-1b17.11 | si:dkey-1b17.11 |
| 4 | MIMAT0001280 | 0.02804 | 100009659 | patl1 | protein associated with topoisomerase II homolog 1 (yeast) |
| 5 | MIMAT0001280 | 0.04673 | 100034415 | si:dkey-6a5.3 | si:dkey-6a5.3 |
| 6 | MIMAT0001280 | 0.03738 | 100170402 | pabpn1l | poly(A) binding protein, nuclear 1, like |
| 7 | MIMAT0001280 | 0.04206 | 100270761 | scpp8 | secretory calcium-binding phosphoprotein 8 |
| 8 | MIMAT0001280 | 0.04673 | 100320152 | steap3 | STEAP family member 3, metalloreductase |
| 9 | MIMAT0001280 | 0.02336 | 246275 | myhz2 | myosin, heavy polypeptide 2, fast muscle specific |
| 10 | MIMAT0001280 | 0.04673 | 30080 | mitfa | microphthalmia-associated transcription factor a |
| 11 | MIMAT0001280 | 0.04673 | 30125 | dvr1 | decapentaplegic and Vg-related 1 |
| 12 | MIMAT0001280 | 0.01869 | 30298 | jak2b | Janus kinase 2b |
| 13 | MIMAT0001280 | 0.01869 | 30586 | dlx6a | distal-less homeobox gene 6a |
| 14 | MIMAT0001280 | 0.01869 | 322927 | asb12a | ankyrin repeat and SOCS box-containing protein 12a |
| 15 | MIMAT0001280 | 0.04206 | 323641 | gpr182 | G protein-coupled receptor 182 |
| 16 | MIMAT0001280 | 0.03738 | 324818 | ctsh | cathepsin H |
| 17 | MIMAT0001280 | 0.00467 | 326977 | atp5l | ATP synthase, H+ transporting, mitochondrial F0 complex, subunit g |
| 18 | MIMAT0001280 | 0.00935 | 327274 | elovl7b | ELOVL family member 7, elongation of long chain fatty acids (yeast) b |
| 19 | MIMAT0001280 | 0.02336 | 334817 | pmp22a | peripheral myelin protein 22a |
| 20 | MIMAT0001280 | 0.01402 | 335623 | pvalb5 | parvalbumin 5 |
| 21 | MIMAT0001280 | 0.03738 | 335958 | wu:fj39g12 | wu:fj39g12 |
| 22 | MIMAT0001280 | 0.02804 | 336283 | mrpl3 | mitochondrial ribosomal protein L3 |
| 23 | MIMAT0001280 | 0.04673 | 352919 | selt1a | selenoprotein T, 1a |
| 24 | MIMAT0001280 | 0.03271 | 352928 | gpx4a | glutathione peroxidase 4a |
| 25 | MIMAT0001280 | 0.04673 | 353247 | tnnc1a | troponin C type 1a (slow) |
| 26 | MIMAT0001280 | 0.04673 | 368200 | piwil1 | piwi-like 1 (Drosophila) |
| 27 | MIMAT0001280 | 0.02804 | 387531 | cpvl | carboxypeptidase, vitellogenic-like |
| 28 | MIMAT0001280 | 0.02804 | 393098 | ctu1 | cytosolic thiouridylase subunit 1 homolog (S. pombe) |
| 29 | MIMAT0001280 | 0.03738 | 393210 | wdr45l | wdr45 like |
| 30 | MIMAT0001280 | 0.03738 | 393339 | slc5a12 | solute carrier family 5 (sodium/glucose cotransporter), member 12 |
| 31 | MIMAT0001280 | 0.01869 | 393611 | c6 | complement component 6 |
| 32 | MIMAT0001280 | 0.02804 | 393647 | dus3l | dihydrouridine synthase 3-like (S. cerevisiae) |
| 33 | MIMAT0001280 | 0.04206 | 393967 | pds5a | PDS5, regulator of cohesion maintenance, homolog A (S. cerevisiae) |
| 34 | MIMAT0001280 | 0.00467 | 394166 | rps21 | ribosomal protein S21 |
| 35 | MIMAT0001280 | 0.02336 | 399662 | mdh1a | malate dehydrogenase 1a, NAD (soluble) |
| 36 | MIMAT0001280 | 0.04673 | 402796 | cdkn3 | cyclin-dependent kinase inhibitor 3 |
| 37 | MIMAT0001280 | 0.03271 | 403015 | si:rp71-45k5.4 | si:rp71-45k5.4 |
| 38 | MIMAT0001280 | 0.03271 | 405869 | pdcd10b | programmed cell death 10b |
| 39 | MIMAT0001280 | 0.01869 | 406294 | glrx5 | glutaredoxin 5 homolog (S. cerevisiae) |
| 40 | MIMAT0001280 | 0.04206 | 406408 | ddost | dolichyl-diphosphooligosaccharide-protein glycosyltransferase |
| 41 | MIMAT0001280 | 0.04206 | 436755 | trappc5 | trafficking protein particle complex 5 |
| 42 | MIMAT0001280 | 0.02336 | 436858 | tcta | T-cell leukemia translocation altered gene |
| 43 | MIMAT0001280 | 0.03271 | 436893 | coq3 | coenzyme Q3 homolog, methyltransferase (yeast) |
| 44 | MIMAT0001280 | 0.01402 | 445095 | tm4sf4 | transmembrane 4 L six family member 4 |
| 45 | MIMAT0001280 | 0.04673 | 445299 | immp2l | IMP2 inner mitochondrial membrane peptidase-like (S. cerevisiae) |
| 46 | MIMAT0001280 | 0.04673 | 449658 | paxip1 | PAX interacting (with transcription-activation domain) protein 1 |
| 47 | MIMAT0001280 | 0.03271 | 450020 | cd151 | CD151 molecule |
| 48 | MIMAT0001280 | 0.02804 | 494090 | bfsp2 | beaded filament structural protein 2, phakinin |
| 49 | MIMAT0001280 | 0.04206 | 541337 | sptssa | serine palmitoyltransferase, small subunit A |
| 50 | MIMAT0001280 | 0.02804 | 541368 | s100t | S100 calcium binding protein T |
| 51 | MIMAT0001280 | 0.04673 | 550272 | alkbh7 | alkB, alkylation repair homolog 7 (E. coli) |
| 52 | MIMAT0001280 | 0.03738 | 550282 | dcxr | dicarbonyl/L-xylulose reductase |
| 53 | MIMAT0001280 | 0.02804 | 553404 | lrrc47 | leucine rich repeat containing 47 |
| 54 | MIMAT0001280 | 0.03271 | 553471 | smarcc1b | SWI/SNF related, matrix associated, actin dependent regulator of chromatin, subfamily c, member 1b |
| 55 | MIMAT0001280 | 0.04206 | 553502 | mis18a | MIS18 kinetochore protein homolog A (S. pombe) |
| 56 | MIMAT0001280 | 0.01402 | 553605 | rab3ip | RAB3A interacting protein (rabin3) |
| 57 | MIMAT0001280 | 0.02804 | 553810 | rmnd1 | required for meiotic nuclear division 1 homolog (S. cerevisiae) |
| 58 | MIMAT0001280 | 0.02804 | 554153 | psma2 | proteasome (prosome, macropain) subunit, alpha type, 2 |
| 59 | MIMAT0001280 | 0.04206 | 554998 | pdia4 | protein disulfide isomerase associated 4 |
| 60 | MIMAT0001280 | 0.04206 | 555452 | taf1 | TAF1 RNA polymerase II, TATA box binding protein (TBP)-associated factor |
| 61 | MIMAT0001280 | 0.04206 | 555455 | bivm | basic, immunoglobulin-like variable motif containing |
| 62 | MIMAT0001280 | 0.04673 | 555679 | scpp5 | secretory calcium-binding phosphoprotein 5 |
| 63 | MIMAT0001280 | 0.02804 | 556426 | rprd2b | regulation of nuclear pre-mRNA domain containing 2b |
| 64 | MIMAT0001280 | 0.01869 | 558036 | lmx1a | LIM homeobox transcription factor 1, alpha |
| 65 | MIMAT0001280 | 0.01402 | 559425 | bida | BH3 interacting domain death agonist |
| 66 | MIMAT0001280 | 0.02804 | 559614 | myom3 | myomesin family, member 3 |
| 67 | MIMAT0001280 | 0.00467 | 559754 | tmprss13a | transmembrane protease, serine 13a |
| 68 | MIMAT0001280 | 0.02336 | 561018 | cmasa | cytidine monophosphate N-acetylneuraminic acid synthetase a |
| 69 | MIMAT0001280 | 0.02804 | 561049 | eif3eb | eukaryotic translation initiation factor 3, subunit E, b |
| 70 | MIMAT0001280 | 0.00935 | 562232 | aimp1 | aminoacyl tRNA synthetase complex-interacting multifunctional protein 1 |
| 71 | MIMAT0001280 | 0.03738 | 562695 | si:dkey-72l14.7 | si:dkey-72l14.7 |
| 72 | MIMAT0001280 | 0.04206 | 563946 | zgc:136930 | zgc:136930 |
| 73 | MIMAT0001280 | 0.04206 | 565201 | hs3st1 | heparan sulfate (glucosamine) 3-O-sulfotransferase 1 |
| 74 | MIMAT0001280 | 0.02336 | 565831 | hsf2bp | heat shock transcription factor 2 binding protein |
| 75 | MIMAT0001280 | 0.04673 | 566165 | glulc | glutamate-ammonia ligase (glutamine synthase) c |
| 76 | MIMAT0001280 | 0.02336 | 567724 | snai3 | snail homolog 3 |
| 77 | MIMAT0001280 | 0.02804 | 569714 | apom | apolipoprotein M |
| 78 | MIMAT0001280 | 0.00935 | 570444 | si:dkey-5n18.1 | si:dkey-5n18.1 |
| 79 | MIMAT0001280 | 0.01869 | 58084 | tnnt3a | troponin T3a, skeletal, fast |
| 80 | MIMAT0001280 | 0.02804 | 58142 | myhz1.1 | myosin, heavy polypeptide 1.1, skeletal muscle |
| 81 | MIMAT0001280 | 0.04673 | 606559 | rnf121 | ring finger protein 121 |
| 82 | MIMAT0001280 | 0.04206 | 64272 | atp1b3b | ATPase, Na+/K+ transporting, beta 3b polypeptide |
| 83 | MIMAT0001280 | 0.03271 | 768298 | faah2b | fatty acid amide hydrolase 2b |
| 84 | MIMAT0001280 | 0.03738 | 777608 | zgc:153126 | zgc:153126 |
| 85 | MIMAT0001280 | 0.02804 | 790924 | usp39 | ubiquitin specific peptidase 39 |
| 86 | MIMAT0001280 | 0.04673 | 791993 | wnt4b | wingless-type MMTV integration site family, member 4b |
| 87 | MIMAT0001280 | 0.04206 | 794053 | il13 | interleukin 13 |
| 88 | MIMAT0001280 | 0.01869 | 798207 | lrrc10 | leucine rich repeat containing 10 |
| 89 | MIMAT0001280 | 0.03738 | 81586 | cldng | claudin g |

Table S5: Significantly (>2.0 fold) down-regulated miRNA in the liver of zebrafish fed and exposed to fluoxetine (540 ng/L) for 7 days and their *in silico* predicted target genes. Target genes are sorted by ascending level of significant likelihood of targeting said gene. Legend: MIMAT0001280 = dre-miR-205; MIMAT0001782 = dre-miR-19a-3p``.

|  | **mirbase accession No** | **p-value** | **GeneID** | **Symbol** | **Description** |
| --- | --- | --- | --- | --- | --- |
| 1 | MIMAT0001280,MIMAT0001782 | 0.01909 | 100001615 | sufu | suppressor of fused homolog (Drosophila) |
| 2 | MIMAT0001280,MIMAT0001782 | 0.03598 | 100001681 | LOC100001681 | potassium voltage-gated channel, Shal-related subfamily, member 1 |
| 3 | MIMAT0001280,MIMAT0001782 | 0.03422 | 100002445 | unc5db | unc-5 homolog Db (C. elegans) |
| 4 | MIMAT0001280,MIMAT0001782 | 0.04344 | 100002993 | ugt5e1 | UDP glucuronosyltransferase 5 family, polypeptide E1 |
| 5 | MIMAT0001280,MIMAT0001782 | 0.02317 | 100004024 | med13a | mediator complex subunit 13a |
| 6 | MIMAT0001280,MIMAT0001782 | 0.00671 | 100004517 | tmem88b | transmembrane protein 88 b |
| 7 | MIMAT0001280,MIMAT0001782 | 0.00834 | 100005529 | dnal4b | dynein, axonemal, light chain 4b |
| 8 | MIMAT0001280,MIMAT0001782 | 0.00597 | 100007906 | parp2 | poly (ADP-ribose) polymerase family, member 2 |
| 9 | MIMAT0001280,MIMAT0001782 | 0.03778 | 100008592 | hs3st3l | heparan sulfate (glucosamine) 3-O-sulfotransferase 3-like |
| 10 | MIMAT0001280,MIMAT0001782 | 0.00750 | 100034390 | si:dkey-234l24.8 | si:dkey-234l24.8 |
| 11 | MIMAT0001280,MIMAT0001782 | 0.03778 | 100034521 | pnrc1 | proline-rich nuclear receptor coactivator 1 |
| 12 | MIMAT0001280,MIMAT0001782 | 0.02461 | 100036767 | plod2 | procollagen-lysine, 2-oxoglutarate 5-dioxygenase 2 |
| 13 | MIMAT0001280,MIMAT0001782 | 0.02040 | 100073327 | zgc:165481 | zgc:165481 |
| 14 | MIMAT0001280,MIMAT0001782 | 0.01540 | 100124610 | timp4a | TIMP metallopeptidase inhibitor 4a |
| 15 | MIMAT0001280,MIMAT0001782 | 0.02922 | 100149956 | rgl3a | ral guanine nucleotide dissociation stimulator-like 3a |
| 16 | MIMAT0001280,MIMAT0001782 | 0.02922 | 100151416 | cdkn1a | cyclin-dependent kinase inhibitor 1A |
| 17 | MIMAT0001280,MIMAT0001782 | 0.02317 | 100286814 | fam101b | family with sequence similarity 101, member B |
| 18 | MIMAT0001280,MIMAT0001782 | 0.04344 | 100301512 | ftr76 | finTRIM family, member 76 |
| 19 | MIMAT0001280,MIMAT0001782 | 0.03251 | 100320028 | plat | plasminogen activator, tissue |
| 20 | MIMAT0001280,MIMAT0001782 | 0.04743 | 100534721 | csmd1 | CUB and Sushi multiple domains 1 |
| 21 | MIMAT0001280,MIMAT0001782 | 0.01659 | 100535855 | ptchd2 | patched domain containing 2 |
| 22 | MIMAT0001280,MIMAT0001782 | 0.04949 | 100538291 | pigg | phosphatidylinositol glycan anchor biosynthesis, class G |
| 23 | MIMAT0001280,MIMAT0001782 | 0.04541 | 192295 | ccna2 | cyclin A2 |
| 24 | MIMAT0001280,MIMAT0001782 | 0.00342 | 192327 | cct3 | chaperonin containing TCP1, subunit 3 (gamma) |
| 25 | MIMAT0001280,MIMAT0001782 | 0.03085 | 192329 | ppp1r10 | protein phosphatase 1, regulatory subunit 10 |
| 26 | MIMAT0001280 | 0.04629 | 246275 | myhz2 | myosin, heavy polypeptide 2, fast muscle specific |
| 27 | MIMAT0001782 | 0.03712 | 30077 | rbp4 | retinol binding protein 4, plasma |
| 28 | MIMAT0001280,MIMAT0001782 | 0.03085 | 30100 | ube2kb | ubiquitin-conjugating enzyme E2Kb (UBC1 homolog, yeast) |
| 29 | MIMAT0001280,MIMAT0001782 | 0.04151 | 30158 | lfng | lunatic fringe homolog |
| 30 | MIMAT0001280,MIMAT0001782 | 0.02764 | 30169 | neurod | neurogenic differentiation |
| 31 | MIMAT0001280,MIMAT0001782 | 0.03085 | 30252 | sema3h | semaphorin 3h |
| 32 | MIMAT0001280 | 0.03712 | 30298 | jak2b | Janus kinase 2b |
| 33 | MIMAT0001280,MIMAT0001782 | 0.03422 | 30313 | ephb3a | eph receptor B3a |
| 34 | MIMAT0001280,MIMAT0001782 | 0.01781 | 30470 | epb4.1l4 | erythrocyte protein band 4.1-like 4 |
| 35 | MIMAT0001280,MIMAT0001782 | 0.04151 | 30488 | ck2a2a | casein kinase 2 alpha 2a |
| 36 | MIMAT0001280 | 0.03712 | 30586 | dlx6a | distal-less homeobox gene 6a |
| 37 | MIMAT0001280,MIMAT0001782 | 0.01426 | 30600 | mid1ip1a | MID1 interacting protein 1a |
| 38 | MIMAT0001280,MIMAT0001782 | 0.02764 | 317738 | elovl6 | ELOVL family member 6, elongation of long chain fatty acids (yeast) |
| 39 | MIMAT0001280,MIMAT0001782 | 0.04541 | 321364 | id2b | inhibitor of DNA binding 2, dominant negative helix-loop-helix protein, b |
| 40 | MIMAT0001280,MIMAT0001782 | 0.01781 | 321541 | slc20a1b | solute carrier family 20, member 1b |
| 41 | MIMAT0001280,MIMAT0001782 | 0.03962 | 322013 | cacng1 | calcium channel, voltage-dependent, gamma subunit 1 |
| 42 | MIMAT0001280,MIMAT0001782 | 0.01659 | 322509 | acta2 | actin, alpha 2, smooth muscle, aorta |
| 43 | MIMAT0001280,MIMAT0001782 | 0.04743 | 322513 | uap1 | UDP-N-acteylglucosamine pyrophosphorylase 1 |
| 44 | MIMAT0001280 | 0.03712 | 322927 | asb12a | ankyrin repeat and SOCS box-containing protein 12a |
| 45 | MIMAT0001280,MIMAT0001782 | 0.03085 | 322953 | dnajc11 | DnaJ (Hsp40) homolog, subfamily C, member 11 |
| 46 | MIMAT0001280,MIMAT0001782 | 0.01781 | 323706 | rtn1a | reticulon 1a |
| 47 | MIMAT0001280,MIMAT0001782 | 0.04949 | 324309 | fstl1b | follistatin-like 1b |
| 48 | MIMAT0001280,MIMAT0001782 | 0.04344 | 325255 | rab18a | RAB18A, member RAS oncogene family |
| 49 | MIMAT0001280,MIMAT0001782 | 0.02611 | 326652 | zgc:73340 | zgc:73340 |
| 50 | MIMAT0001280,MIMAT0001782 | 0.04743 | 326954 | tecrb | trans-2,3-enoyl-CoA reductase b |
| 51 | MIMAT0001280 | 0.00935 | 326977 | atp5l | ATP synthase, H+ transporting, mitochondrial F0 complex, subunit g |
| 52 | MIMAT0001280,MIMAT0001782 | 0.02176 | 327167 | mynn | myoneurin |
| 53 | MIMAT0001280,MIMAT0001782 | 0.00342 | 327246 | dusp7 | dual specificity phosphatase 7 |
| 54 | MIMAT0001280 | 0.01865 | 327274 | elovl7b | ELOVL family member 7, elongation of long chain fatty acids (yeast) b |
| 55 | MIMAT0001280,MIMAT0001782 | 0.01781 | 327395 | ndrg3b | N-myc downstream regulated family member 3b |
| 56 | MIMAT0001782 | 0.03712 | 334557 | zgc:173742 | zgc:173742 |
| 57 | MIMAT0001280 | 0.04629 | 334817 | pmp22a | peripheral myelin protein 22a |
| 58 | MIMAT0001280,MIMAT0001782 | 0.01659 | 334827 | wdr13 | WD repeat domain 13 |
| 59 | MIMAT0001280,MIMAT0001782 | 0.02176 | 334932 | mdm4 | transformed 3T3 cell double minute 4 homolog (mouse) |
| 60 | MIMAT0001280 | 0.02791 | 335623 | pvalb5 | parvalbumin 5 |
| 61 | MIMAT0001280,MIMAT0001782 | 0.02922 | 336121 | calm2a | calmodulin 2a (phosphorylase kinase, delta) |
| 62 | MIMAT0001280,MIMAT0001782 | 0.03598 | 336281 | vamp2 | vesicle-associated membrane protein 2 |
| 63 | MIMAT0001782,MIMAT0001280 | 0.00290 | 336695 | samsn1a | SAM domain, SH3 domain and nuclear localisation signals, 1a |
| 64 | MIMAT0001280,MIMAT0001782 | 0.04151 | 368476 | pcdh15a | protocadherin 15a |
| 65 | MIMAT0001280,MIMAT0001782 | 0.04151 | 368672 | atad1a | ATPase family, AAA domain containing 1a |
| 66 | MIMAT0001280,MIMAT0001782 | 0.02040 | 373118 | casp2 | caspase 2, apoptosis-related cysteine protease |
| 67 | MIMAT0001280,MIMAT0001782 | 0.01110 | 373882 | mmp14a | matrix metalloproteinase 14a (membrane-inserted) |
| 68 | MIMAT0001280,MIMAT0001782 | 0.01659 | 386589 | hopx | HOP homeobox |
| 69 | MIMAT0001280,MIMAT0001782 | 0.04151 | 386920 | hist2h2l | histone 2, H2, like |
| 70 | MIMAT0001280,MIMAT0001782 | 0.00750 | 393203 | prc1b | protein regulator of cytokinesis 1b |
| 71 | MIMAT0001280,MIMAT0001782 | 0.03962 | 393445 | ube3c | ubiquitin protein ligase E3C |
| 72 | MIMAT0001280,MIMAT0001782 | 0.04151 | 393464 | tmem68 | transmembrane protein 68 |
| 73 | MIMAT0001280,MIMAT0001782 | 0.03085 | 393467 | zgc:66127 | zgc:66127 |
| 74 | MIMAT0001280 | 0.03712 | 393611 | c6 | complement component 6 |
| 75 | MIMAT0001280,MIMAT0001782 | 0.01426 | 393679 | cib2 | calcium and integrin binding family member 2 |
| 76 | MIMAT0001280,MIMAT0001782 | 0.03085 | 393775 | clu | clusterin |
| 77 | MIMAT0001280,MIMAT0001782 | 0.02611 | 394069 | ebag9 | estrogen receptor binding site associated, antigen, 9 |
| 78 | MIMAT0001280,MIMAT0001782 | 0.01781 | 394117 | rab1ba | zRAB1B, member RAS oncogene family a |
| 79 | MIMAT0001280 | 0.00935 | 394166 | rps21 | ribosomal protein S21 |
| 80 | MIMAT0001280 | 0.04629 | 399662 | mdh1a | malate dehydrogenase 1a, NAD (soluble) |
| 81 | MIMAT0001280,MIMAT0001782 | 0.03422 | 402816 | pou4f2 | POU domain, class 4, transcription factor 2 |
| 82 | MIMAT0001280,MIMAT0001782 | 0.00834 | 402929 | evlb | Enah/Vasp-like b |
| 83 | MIMAT0001280,MIMAT0001782 | 0.02611 | 402976 | si:dkey-194e6.1 | si:dkey-194e6.1 |
| 84 | MIMAT0001280,MIMAT0001782 | 0.03251 | 403028 | slc31a1 | solute carrier family 31 (copper transporters), member 1 |
| 85 | MIMAT0001280,MIMAT0001782 | 0.04151 | 404208 | myf6 | myogenic factor 6 |
| 86 | MIMAT0001280,MIMAT0001782 | 0.02461 | 404630 | zc3h10 | zinc finger CCCH-type containing 10 |
| 87 | MIMAT0001280,MIMAT0001782 | 0.01659 | 405766 | b3gnt2a | UDP-GlcNAc:betaGal beta-1,3-N-acetylglucosaminyltransferase 2a |
| 88 | MIMAT0001280,MIMAT0001782 | 0.00671 | 405870 | ucmab | upper zone of growth plate and cartilage matrix associated b |
| 89 | MIMAT0001280,MIMAT0001782 | 0.04541 | 406202 | heg | heart of glass |
| 90 | MIMAT0001280,MIMAT0001782 | 0.04344 | 406205 | mnx2a | motor neuron and pancreas homeobox 2a |
| 91 | MIMAT0001280,MIMAT0001782 | 0.04344 | 406274 | klhl20 | kelch-like 20 (Drosophila) |
| 92 | MIMAT0001280 | 0.03712 | 406294 | glrx5 | glutaredoxin 5 homolog (S. cerevisiae) |
| 93 | MIMAT0001280,MIMAT0001782 | 0.02611 | 406347 | slc35e1 | solute carrier family 35, member E1 |
| 94 | MIMAT0001280,MIMAT0001782 | 0.03085 | 406361 | dok1b | docking protein 1b |
| 95 | MIMAT0001280,MIMAT0001782 | 0.02461 | 406372 | wdr5 | WD repeat domain 5 |
| 96 | MIMAT0001280,MIMAT0001782 | 0.02922 | 406587 | rpf2 | ribosome production factor 2 homolog (S. cerevisiae) |
| 97 | MIMAT0001280,MIMAT0001782 | 0.04541 | 406596 | socs3b | suppressor of cytokine signaling 3b |
| 98 | MIMAT0001280,MIMAT0001782 | 0.02611 | 406853 | creb3l3l | cAMP responsive element binding protein 3-like 3 like |
| 99 | MIMAT0001280,MIMAT0001782 | 0.04743 | 407076 | chst12a | carbohydrate (chondroitin 4) sulfotransferase 12a |
| 100 | MIMAT0001280,MIMAT0001782 | 0.02317 | 407664 | hbegfb | heparin-binding EGF-like growth factor b |
| 101 | MIMAT0001280,MIMAT0001782 | 0.01659 | 407688 | sft2d3 | SFT2 domain containing 3 |
| 102 | MIMAT0001280,MIMAT0001782 | 0.03251 | 415220 | mpdu1b | mannose-P-dolichol utilization defect 1b |
| 103 | MIMAT0001280,MIMAT0001782 | 0.03422 | 431724 | impdh1a | inosine 5'-phosphate dehydrogenase 1a |
| 104 | MIMAT0001280,MIMAT0001782 | 0.02611 | 432387 | pkd2 | polycystic kidney disease 2 |
| 105 | MIMAT0001280,MIMAT0001782 | 0.03422 | 436644 | eef1a2 | eukaryotic translation elongation factor 1 alpha 2 |
| 106 | MIMAT0001280,MIMAT0001782 | 0.04151 | 436670 | gng7 | guanine nucleotide binding protein (G protein), gamma 7 |
| 107 | MIMAT0001280,MIMAT0001782 | 0.03085 | 436679 | nr4a2b | nuclear receptor subfamily 4, group A, member 2b |
| 108 | MIMAT0001280,MIMAT0001782 | 0.04743 | 436697 | slc48a1a | solute carrier family 48 (heme transporter), member 1a |
| 109 | MIMAT0001280 | 0.04629 | 436858 | tcta | T-cell leukemia translocation altered gene |
| 110 | MIMAT0001280,MIMAT0001782 | 0.00597 | 437006 | nxph1 | neurexophilin 1 |
| 111 | MIMAT0001280,MIMAT0001782 | 0.04541 | 442923 | oxct1a | 3-oxoacid CoA transferase 1a |
| 112 | MIMAT0001280,MIMAT0001782 | 0.01316 | 445027 | sumo2b | SMT3 suppressor of mif two 3 homolog 2b |
| 113 | MIMAT0001280,MIMAT0001782 | 0.04151 | 445031 | ppp2r4 | protein phosphatase 2A activator, regulatory subunit 4 |
| 114 | MIMAT0001280 | 0.02791 | 445095 | tm4sf4 | transmembrane 4 L six family member 4 |
| 115 | MIMAT0001280,MIMAT0001782 | 0.01426 | 445119 | traf3 | TNF receptor-associated factor 3 |
| 116 | MIMAT0001280,MIMAT0001782 | 0.03085 | 445133 | kctd15b | potassium channel tetramerisation domain containing 15b |
| 117 | MIMAT0001280,MIMAT0001782 | 0.01540 | 445137 | fam151b | family with sequence similarity 151, member B |
| 118 | MIMAT0001280,MIMAT0001782 | 0.04151 | 445189 | optc | opticin |
| 119 | MIMAT0001280,MIMAT0001782 | 0.03778 | 445310 | tmem179 | transmembrane protein 179 |
| 120 | MIMAT0001280,MIMAT0001782 | 0.02922 | 445320 | hspbap1 | hspb associated protein 1 |
| 121 | MIMAT0001280,MIMAT0001782 | 0.03085 | 445393 | ppp1r12a | protein phosphatase 1, regulatory (inhibitor) subunit 12A |
| 122 | MIMAT0001280,MIMAT0001782 | 0.01211 | 445403 | uspl1 | ubiquitin specific peptidase like 1 |
| 123 | MIMAT0001280,MIMAT0001782 | 0.00399 | 445492 | flad1 | FAD1 flavin adenine dinucleotide synthetase homolog (S. cerevisiae) |
| 124 | MIMAT0001280,MIMAT0001782 | 0.03251 | 446116 | rnf113a | ring finger protein 113A |
| 125 | MIMAT0001280,MIMAT0001782 | 0.03962 | 447799 | nhsl1b | NHS-like 1b |
| 126 | MIMAT0001280,MIMAT0001782 | 0.02922 | 447941 | apip | APAF1 interacting protein |
| 127 | MIMAT0001280,MIMAT0001782 | 0.02764 | 449538 | opcml | opioid binding protein/cell adhesion molecule-like |
| 128 | MIMAT0001280,MIMAT0001782 | 0.03085 | 449546 | ric8a | resistance to inhibitors of cholinesterase 8 homolog A |
| 129 | MIMAT0001280,MIMAT0001782 | 0.01781 | 449774 | arl2 | ADP-ribosylation factor-like 2 |
| 130 | MIMAT0001280,MIMAT0001782 | 0.00597 | 450009 | mrps16 | mitochondrial ribosomal protein S16 |
| 131 | MIMAT0001280,MIMAT0001782 | 0.04151 | 474349 | magi1b | membrane associated guanylate kinase, WW and PDZ domain containing 1b |
| 132 | MIMAT0001280,MIMAT0001782 | 0.01110 | 492358 | cx28.9 | connexin 28.9 |
| 133 | MIMAT0001280,MIMAT0001782 | 0.02611 | 492765 | xkrx | XK, Kell blood group complex subunit-related, X-linked |
| 134 | MIMAT0001280,MIMAT0001782 | 0.03778 | 494077 | tsta3 | tissue specific transplantation antigen P35B |
| 135 | MIMAT0001280,MIMAT0001782 | 0.02922 | 503600 | cltcb | clathrin, heavy polypeptide b (Hc) |
| 136 | MIMAT0001280,MIMAT0001782 | 0.04949 | 503743 | smtnl1 | smoothelin-like 1 |
| 137 | MIMAT0001280,MIMAT0001782 | 0.03422 | 541365 | zdhhc2 | zinc finger, DHHC-type containing 2 |
| 138 | MIMAT0001280,MIMAT0001782 | 0.04541 | 541372 | phf23a | PHD finger protein 23a |
| 139 | MIMAT0001280,MIMAT0001782 | 0.03422 | 541556 | rbm39b | RNA binding motif protein 39b |
| 140 | MIMAT0001280,MIMAT0001782 | 0.01540 | 550324 | kctd12b | potassium channel tetramerisation domain containing 12b |
| 141 | MIMAT0001280,MIMAT0001782 | 0.04743 | 550327 | pcyt1aa | phosphate cytidylyltransferase 1, choline, alpha a |
| 142 | MIMAT0001280,MIMAT0001782 | 0.04344 | 550393 | praf2 | PRA1 domain family, member 2 |
| 143 | MIMAT0001280,MIMAT0001782 | 0.01909 | 553431 | camkvl | CaM kinase-like vesicle-associated, like |
| 144 | MIMAT0001280,MIMAT0001782 | 0.02317 | 553529 | bloc1s3 | biogenesis of lysosomal organelles complex-1, subunit 3 |
| 145 | MIMAT0001280,MIMAT0001782 | 0.01014 | 553544 | nsmce4a | non-SMC element 4 homolog A (S. cerevisiae) |
| 146 | MIMAT0001280 | 0.02791 | 553605 | rab3ip | RAB3A interacting protein (rabin3) |
| 147 | MIMAT0001280,MIMAT0001782 | 0.02611 | 553788 | map3k7 | mitogen activated protein kinase kinase kinase 7 |
| 148 | MIMAT0001280,MIMAT0001782 | 0.04344 | 553816 | inhbab | inhibin, beta Ab |
| 149 | MIMAT0001280,MIMAT0001782 | 0.04344 | 555223 | tspear | thrombospondin-type laminin G domain and EAR repeats |
| 150 | MIMAT0001280,MIMAT0001782 | 0.03778 | 555385 | siae | sialic acid acetylesterase |
| 151 | MIMAT0001280,MIMAT0001782 | 0.02611 | 556043 | aftpha | aftiphilin a |
| 152 | MIMAT0001280,MIMAT0001782 | 0.00342 | 556315 | gxylt1b | glucoside xylosyltransferase 1b |
| 153 | MIMAT0001280,MIMAT0001782 | 0.03778 | 556591 | prkchb | protein kinase C, eta, b |
| 154 | MIMAT0001280,MIMAT0001782 | 0.03422 | 556642 | trub1 | TruB pseudouridine (psi) synthase homolog 1 (E. coli) |
| 155 | MIMAT0001280,MIMAT0001782 | 0.01781 | 556728 | crfb8 | cytokine receptor family member b8 |
| 156 | MIMAT0001280,MIMAT0001782 | 0.02764 | 557065 | pxk | PX domain containing serine/threonine kinase |
| 157 | MIMAT0001280,MIMAT0001782 | 0.03085 | 557552 | zgc:153031 | zgc:153031 |
| 158 | MIMAT0001280,MIMAT0001782 | 0.04151 | 557693 | rapgef2 | Rap guanine nucleotide exchange factor (GEF) 2 |
| 159 | MIMAT0001280,MIMAT0001782 | 0.04541 | 557795 | col8a1a | collagen, type VIII, alpha 1a |
| 160 | MIMAT0001280,MIMAT0001782 | 0.01426 | 557812 | fam184b | family with sequence similarity 184, member B |
| 161 | MIMAT0001280,MIMAT0001782 | 0.04151 | 557878 | si:ch211-247j6.1 | si:ch211-247j6.1 |
| 162 | MIMAT0001280,MIMAT0001782 | 0.02317 | 557997 | olfml2ba | olfactomedin-like 2Ba |
| 163 | MIMAT0001280,MIMAT0001782 | 0.01426 | 557999 | tdrd5 | tudor domain containing 5 |
| 164 | MIMAT0001280 | 0.03712 | 558036 | lmx1a | LIM homeobox transcription factor 1, alpha |
| 165 | MIMAT0001782 | 0.03712 | 558042 | ppil1 | peptidylprolyl isomerase (cyclophilin)-like 1 |
| 166 | MIMAT0001280,MIMAT0001782 | 0.03598 | 558271 | fscn1a | fascin homolog 1, actin-bundling protein a (Strongylocentrotus purpuratus) |
| 167 | MIMAT0001280,MIMAT0001782 | 0.01316 | 558335 | abca12 | ATP-binding cassette, sub-family A (ABC1), member 12 |
| 168 | MIMAT0001280,MIMAT0001782 | 0.03251 | 558950 | grasp | GRP1 (general receptor for phosphoinositides 1)-associated scaffold protein |
| 169 | MIMAT0001280,MIMAT0001782 | 0.02611 | 559018 | clul1 | clusterin-like 1 (retinal) |
| 170 | MIMAT0001280 | 0.02791 | 559425 | bida | BH3 interacting domain death agonist |
| 171 | MIMAT0001280,MIMAT0001782 | 0.03778 | 559479 | frs2a | fibroblast growth factor receptor substrate 2a |
| 172 | MIMAT0001280 | 0.00935 | 559754 | tmprss13a | transmembrane protease, serine 13a |
| 173 | MIMAT0001280,MIMAT0001782 | 0.02922 | 559796 | bhlhe23 | basic helix-loop-helix family, member e23 |
| 174 | MIMAT0001280,MIMAT0001782 | 0.04151 | 559995 | unc93a | unc-93 homolog A (C. elegans) |
| 175 | MIMAT0001280 | 0.04629 | 561018 | cmasa | cytidine monophosphate N-acetylneuraminic acid synthetase a |
| 176 | MIMAT0001280,MIMAT0001782 | 0.03598 | 561144 | jazf1a | JAZF zinc finger 1a |
| 177 | MIMAT0001280,MIMAT0001782 | 0.03778 | 561606 | atoh8 | atonal homolog 8 |
| 178 | MIMAT0001280,MIMAT0001782 | 0.04541 | 561787 | slc4a1b | solute carrier family 4, anion exchanger, member 1b |
| 179 | MIMAT0001280,MIMAT0001782 | 0.01659 | 562052 | kif14 | kinesin family member 14 |
| 180 | MIMAT0001280,MIMAT0001782 | 0.02611 | 562188 | myo5aa | myosin VAa |
| 181 | MIMAT0001280 | 0.01865 | 562232 | aimp1 | aminoacyl tRNA synthetase complex-interacting multifunctional protein 1 |
| 182 | MIMAT0001280,MIMAT0001782 | 0.03422 | 562264 | arhgef18b | rho/rac guanine nucleotide exchange factor (GEF) 18b |
| 183 | MIMAT0001280,MIMAT0001782 | 0.03778 | 562607 | tnpo1 | transportin 1 |
| 184 | MIMAT0001280,MIMAT0001782 | 0.01316 | 562762 | slc39a4 | solute carrier family 39 (zinc transporter), member 4 |
| 185 | MIMAT0001280,MIMAT0001782 | 0.01211 | 563179 | zp3c | zona pellucida glycoprotein 3c |
| 186 | MIMAT0001280,MIMAT0001782 | 0.04743 | 563498 | enpp5 | ectonucleotide pyrophosphatase/phosphodiesterase 5 |
| 187 | MIMAT0001280,MIMAT0001782 | 0.04151 | 564088 | pik3r4 | phosphoinositide-3-kinase, regulatory subunit 4 |
| 188 | MIMAT0001280,MIMAT0001782 | 0.02922 | 564478 | ip6k1 | inositol hexakisphosphate kinase 1 |
| 189 | MIMAT0001280,MIMAT0001782 | 0.03085 | 564612 | tmem194b | transmembrane protein 194B |
| 190 | MIMAT0001280,MIMAT0001782 | 0.01014 | 565482 | hdac6 | histone deacetylase 6 |
| 191 | MIMAT0001280,MIMAT0001782 | 0.03422 | 565579 | tspan3a | tetraspanin 3a |
| 192 | MIMAT0001280,MIMAT0001782 | 0.03598 | 565743 | pld2 | phospholipase D2 |
| 193 | MIMAT0001280 | 0.04629 | 565831 | hsf2bp | heat shock transcription factor 2 binding protein |
| 194 | MIMAT0001782 | 0.04629 | 565869 | klf9 | Kruppel-like factor 9 |
| 195 | MIMAT0001280,MIMAT0001782 | 0.03085 | 566120 | grk7a | G-protein-coupled receptor kinase 7a |
| 196 | MIMAT0001280,MIMAT0001782 | 0.02922 | 566471 | pigr | polymeric immunoglobulin receptor |
| 197 | MIMAT0001280,MIMAT0001782 | 0.04151 | 566657 | si:dkey-110k5.6 | si:dkey-110k5.6 |
| 198 | MIMAT0001280,MIMAT0001782 | 0.04151 | 567545 | klhdc5 | kelch domain containing 5 |
| 199 | MIMAT0001280 | 0.04629 | 567724 | snai3 | snail homolog 3 |
| 200 | MIMAT0001280,MIMAT0001782 | 0.04743 | 567938 | pik3c2b | phosphoinositide-3-kinase, class 2, beta polypeptide |
| 201 | MIMAT0001280,MIMAT0001782 | 0.04151 | 568117 | spns2 | spinster homolog 2 (Drosophila) |
| 202 | MIMAT0001280,MIMAT0001782 | 0.04344 | 568430 | gpr37b | G protein-coupled receptor 37b |
| 203 | MIMAT0001280,MIMAT0001782 | 0.02461 | 568603 | fam13b | family with sequence similarity 13, member B |
| 204 | MIMAT0001280,MIMAT0001782 | 0.03085 | 568646 | kcnq5b | potassium voltage-gated channel, KQT-like subfamily, member 5b |
| 205 | MIMAT0001280,MIMAT0001782 | 0.01781 | 568666 | atp13a2 | ATPase type 13A2 |
| 206 | MIMAT0001280,MIMAT0001782 | 0.01014 | 568760 | si:dkey-110c1.10 | si:dkey-110c1.10 |
| 207 | MIMAT0001280,MIMAT0001782 | 0.01909 | 568838 | slka | STE20-like kinase a |
| 208 | MIMAT0001280,MIMAT0001782 | 0.01426 | 569041 | suv420h2 | suppressor of variegation 4-20 homolog 2 (Drosophila) |
| 209 | MIMAT0001280,MIMAT0001782 | 0.02764 | 569334 | tbc1d30 | TBC1 domain family, member 30 |
| 210 | MIMAT0001280,MIMAT0001782 | 0.04151 | 569354 | brd2b | bromodomain containing 2b |
| 211 | MIMAT0001280 | 0.01865 | 570444 | si:dkey-5n18.1 | si:dkey-5n18.1 |
| 212 | MIMAT0001280,MIMAT0001782 | 0.03778 | 570745 | letm1 | leucine zipper-EF-hand containing transmembrane protein 1 |
| 213 | MIMAT0001280,MIMAT0001782 | 0.01014 | 571528 | armc8 | armadillo repeat containing 8 |
| 214 | MIMAT0001280,MIMAT0001782 | 0.01211 | 571727 | baz1b | bromodomain adjacent to zinc finger domain, 1B |
| 215 | MIMAT0001280,MIMAT0001782 | 0.04151 | 573655 | rhof | ras homolog gene family, member F |
| 216 | MIMAT0001280,MIMAT0001782 | 0.01426 | 57922 | celf3a | cugbp, Elav-like family member 3a |
| 217 | MIMAT0001280,MIMAT0001782 | 0.04151 | 58013 | noc2l | nucleolar complex associated 2 homolog (S. cerevisiae) |
| 218 | MIMAT0001280,MIMAT0001782 | 0.03422 | 58081 | baxa | bcl2-associated X protein, a |
| 219 | MIMAT0001280 | 0.03712 | 58084 | tnnt3a | troponin T3a, skeletal, fast |
| 220 | MIMAT0001280,MIMAT0001782 | 0.00921 | 606498 | pabpc1a | poly A binding protein, cytoplasmic 1 a |
| 221 | MIMAT0001280,MIMAT0001782 | 0.04949 | 613246 | mpi | mannose phosphate isomerase |
| 222 | MIMAT0001280,MIMAT0001782 | 0.02922 | 619520 | ihha | Indian hedgehog homolog a |
| 223 | MIMAT0001280,MIMAT0001782 | 0.03778 | 678558 | mtmr7a | myotubularin related protein 7a |
| 224 | MIMAT0001280,MIMAT0001782 | 0.03962 | 678612 | zgc:136892 | zgc:136892 |
| 225 | MIMAT0001280,MIMAT0001782 | 0.01211 | 724007 | stra6 | stimulated by retinoic acid gene 6 homolog (mouse) |
| 226 | MIMAT0001280,MIMAT0001782 | 0.02764 | 767643 | eml1 | echinoderm microtubule associated protein like 1 |
| 227 | MIMAT0001280,MIMAT0001782 | 0.02040 | 767681 | btr12 | bloodthirsty-related gene family, member 12 |
| 228 | MIMAT0001280,MIMAT0001782 | 0.02176 | 767761 | znrf2b | zinc and ring finger 2b |
| 229 | MIMAT0001280,MIMAT0001782 | 0.03251 | 767809 | runx1t1 | runt-related transcription factor 1; translocated to, 1 (cyclin D-related) |
| 230 | MIMAT0001280,MIMAT0001782 | 0.03962 | 768158 | pm20d1.2 | peptidase M20 domain containing 1, tandem duplicate 2 |
| 231 | MIMAT0001280,MIMAT0001782 | 0.03422 | 777747 | zgc:153615 | zgc:153615 |
| 232 | MIMAT0001280,MIMAT0001782 | 0.01014 | 791211 | mrps2 | mitochondrial ribosomal protein S2 |
| 233 | MIMAT0001280,MIMAT0001782 | 0.02922 | 792197 | bgnb | biglycan b |
| 234 | MIMAT0001280,MIMAT0001782 | 0.02611 | 792928 | tgfbr1b | transforming growth factor, beta receptor 1 b |
| 235 | MIMAT0001280,MIMAT0001782 | 0.02176 | 793480 | kcnk1a | potassium channel, subfamily K, member 1a |
| 236 | MIMAT0001280,MIMAT0001782 | 0.01540 | 794666 | itpr3 | inositol 1,4,5-triphosphate receptor, type 3 |
| 237 | MIMAT0001280,MIMAT0001782 | 0.03778 | 795522 | rbl1 | retinoblastoma-like 1 (p107) |
| 238 | MIMAT0001280,MIMAT0001782 | 0.04743 | 797269 | abcb11a | ATP-binding cassette, sub-family B (MDR/TAP), member 11a |
| 239 | MIMAT0001280,MIMAT0001782 | 0.03251 | 797304 | smc6 | structural maintenance of chromosomes 6 |
| 240 | MIMAT0001280 | 0.03712 | 798207 | lrrc10 | leucine rich repeat containing 10 |
| 241 | MIMAT0001280,MIMAT0001782 | 0.02922 | 798766 | zgc:153153 | zgc:153153 |
| 242 | MIMAT0001280,MIMAT0001782 | 0.03085 | 799387 | galntl1 | UDP-N-acetyl-alpha-D-galactosamine:polypeptide N-acetylgalactosaminyltransferase-like 1 |
| 243 | MIMAT0001280,MIMAT0001782 | 0.04743 | 81587 | cldnh | claudin h |
| 244 | MIMAT0001280,MIMAT0001782 | 0.01110 | 81595 | bcl7bb | B-cell CLL/lymphoma 7B, b |
| 245 | MIMAT0001280,MIMAT0001782 | 0.01211 | 83496 | bambia | BMP and activin membrane-bound inhibitor (Xenopus laevis) homolog a |
| 246 | MIMAT0001280,MIMAT0001782 | 0.04743 | 83773 | dmd | dystrophin |
| 247 | MIMAT0001280,MIMAT0001782 | 0.00921 | 83910 | bag6 | BCL2-associated athanogene 6 |

Figure S1: Relative transcript abundance of slc6a4a and slc6a4b to ef1α across all treatments. Values for are presented as the average ± S.E.M. (n=4). An * indicates a significant difference between the relative expression of one isoform versus the other in a given treatment as determined by a Student’s t-test (p<0.05). There were no significant differences across treatment groups.


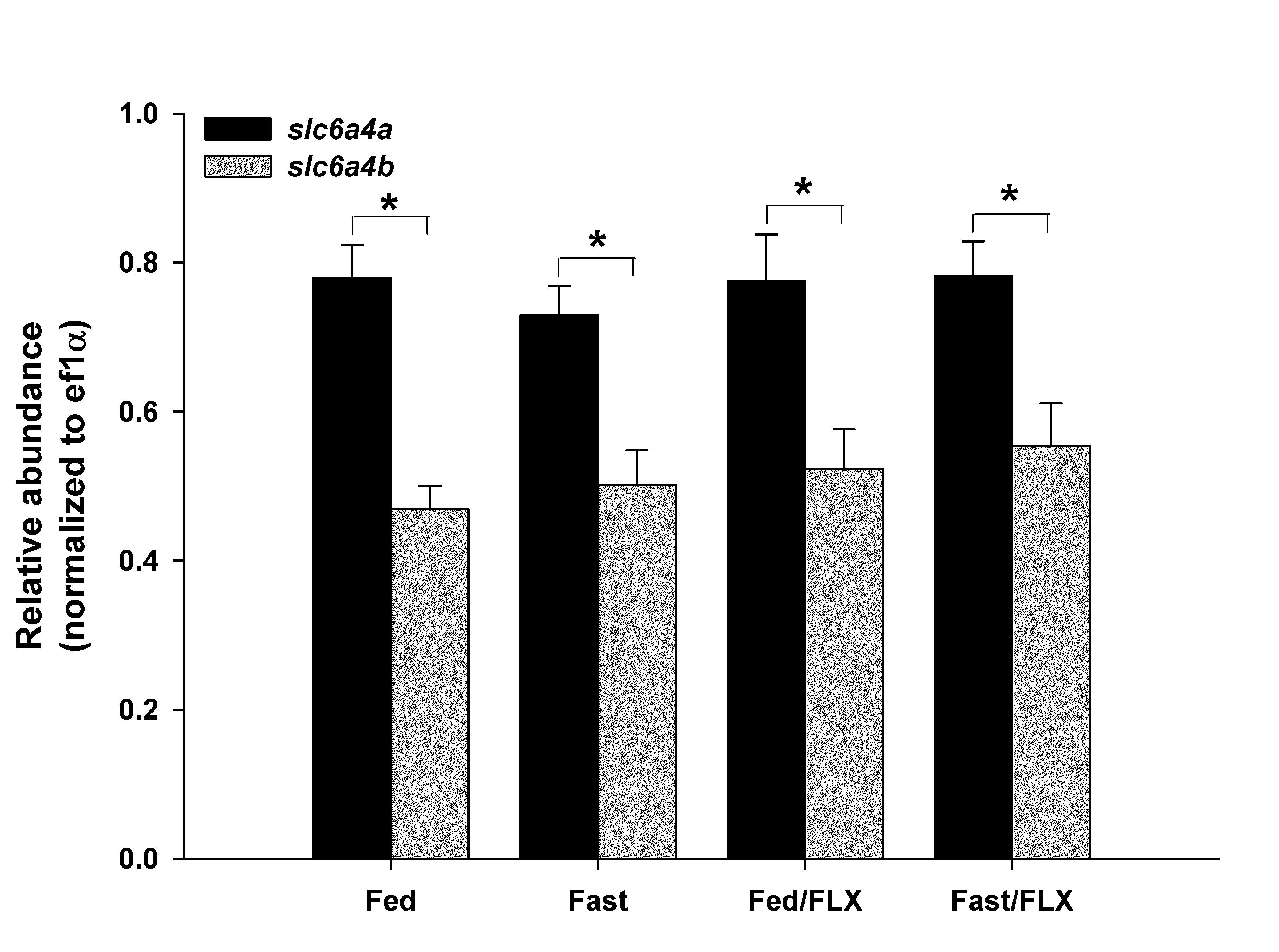

Supplement: File S1 — (DOCX) [file pone.0095351.s001.docx]
